# The alkoxy radical polymerization of *N*-vinylpyrrolidone in organic solvents: theoretical insight into the mechanism and kinetics†

**DOI:** 10.1039/d3ra03820c

**Published:** 2023-08-04

**Authors:** Quan V. Vo, Truong Le Bich Tram, Loc Phuoc Hoang, Nguyen Thi Hoa, Adam Mechler

**Affiliations:** a The University of Danang—University of Technology and Education Danang 550000 Vietnam vvquan@ute.udn.vn vovanquan1980@gmail.com; b Department of Science-Technology and Environment, The University of Danang Danang 550000 Vietnam tlbtram@ac.udn.vn; c Quang Tri Teacher Training College Dong Ha Quang Tri 520000 Vietnam loc_hp@qtttc.edu.vn; d Department of Biochemistry and Chemistry, La Trobe University Victoria 3086 Australia

## Abstract

Poly(*N*-vinylpyrrolidone) (PVP) is a polymer with many applications in cosmetic, pharmaceutical, and biomedical formulations due to its minimal toxicity. PVP can be synthesized through radical polymerization in organic solvents; this well-known industrial process is thoroughly characterized experimentally, however, quantum chemical modeling of the process is scarce: the mechanism and kinetics have not been thoroughly investigated yet. In this work, the mechanism and kinetics of the alkoxy radical polymerization of *N*-vinylpyrrolidone in organic solvents, namely isopropanol (IP) and toluene (TL), were successfully modeled by computational chemistry. The initiator radicals di-*tert*-butyl peroxide (TBO˙) and dicumyl peroxide (CMO˙) as well as the solvents isopropanol and toluene, were shown to be capable of assisting in the initiation reactions. The rate constant was influenced by the combination of initiators and solvent and the values of the rate constant of propagation were approximately 10^1^–10^3^ M^−1^ s^−1^. The radical polymerization of NVP with dicumyl peroxide as an initiator was comparable to that of di-*tert*-butyl peroxide in all of the examined organic solvents, whereas the solvents had less of an effect.

## Abstract 2

The calculations validated the radical polymerization of NVP in organic solvents with propagation rate constants of 10^1^–10^3^ M^−1^ s^−1^.

## Graphical abstract

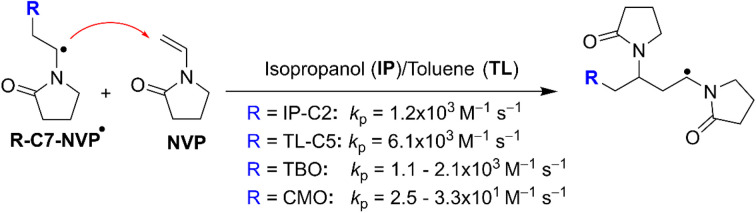

## 1. Introduction

In cosmetic, pharmaceutical, and biomedical formulations, poly(*N*-vinylpyrrolidone) (PVP) is frequently used because of its biocompatibility, biodegradability, potent complexing ability, and superior film-forming qualities.^1^ It is a nonionic, amorphous polymer that is soluble in both organic solvents and water.^2,3^ In medical formulations, it can be found in a wide range of granules, tablets, soft gelatin capsules, hydrogels, films, palettes, and other medical device coatings.^2^

**Fig. 1:**
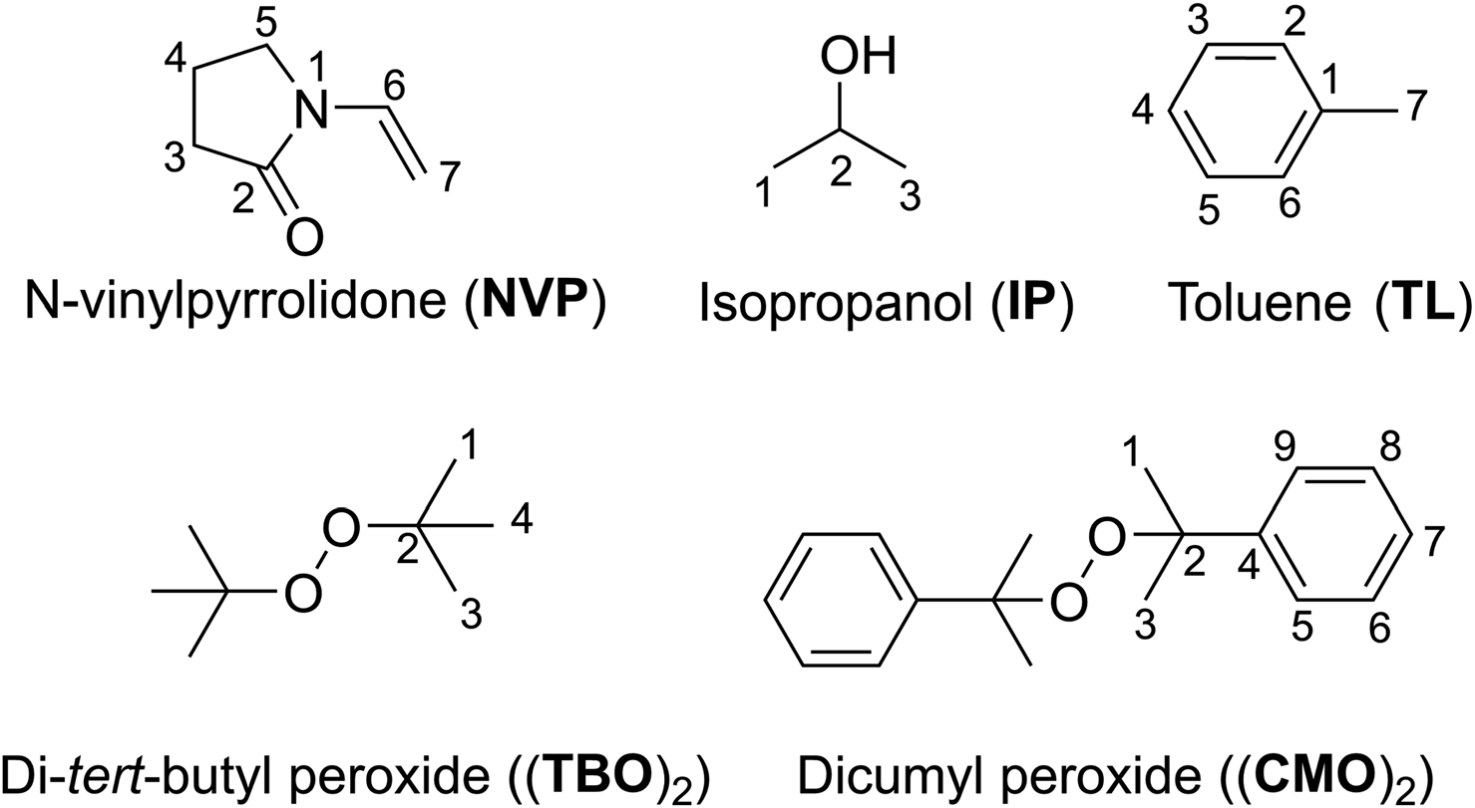
Structure and numbering of NVP, (TBO)_2_, (CMO)_2,_IP and TL.

*N*-Vinylpyrrolidone (NVP, Fig. 1) is typically polymerized in an aqueous solution with hydrogen peroxide as initiator, or in organic solvents such as isopropanol (IP) or toluene (TL) with organic peroxides such as di-*tert*-butyl peroxide (TBO)_2_ or dicumyl peroxide (CMO)_2_ as initiators (Fig. 1).^4–6^ The radicals in the organic solvent, such as alcohols or toluene, act as the reaction's initiators when polymerization is carried out with organic peroxides like (TBO)_2_ or (CMO)_2_. It was discovered that the PVP made in organic solutions was more stable, and no pyrrolidone impurity production was seen, unlike when polymerization was carried out in aqueous solvents with H_2_O_2_ as an initiator.^5^ The polymerization of NVP in polar media by radicals *i.e.* HO˙ garnered some attention in the literature,^7–16^ where the mechanism and kinetics have been investigated.^10,11,16^ However, the alkoxy radical polymerization in organic solvents was not studied with the same fervor, despite of the fact that the PVP made in organic solutions was more stable and no pyrrolidone impurity production was seen.^4,5^

In the field of radical reactions and polymerizations, the *in silico* approach has gained popularity recently as a technique for analyzing the kinetics and mechanism of radical processes. While using the least amount of resources and time possible, new techniques and procedures create dependable data.^17–30^ In this study, we use a well-established method based on quantum chemistry,^16,31,32^ to investigate the alkoxy radical (TBO˙ and CMO˙) polymerization of *N*-vinylpyrrolidone in IP and TL.

## 2. Computational details

The kinetic calculations were performed using the quantum mechanics-based test for the overall free radical scavenging activity (QM-ORSA) technique,^31^ which is directly applicable given the chemical analogy between all radical reactions.^17,33,34^

The rate constant (*k*) was determined by applying eqn (1) to the 1 M standard state at 298.15 K and the transition state theory (TST).^35–39^1
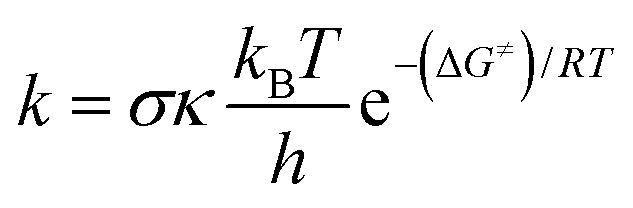
where *σ* is the reaction symmetry number,^40,41^*κ* stands for tunneling corrections that were calculated using Eckart barrier,^42^*k*_B_ is the Boltzmann constant, *h* is the Planck constant and Δ*G*^≠^ is Gibbs free energy of activation.

Those rate constants near the diffusion limit were modified.^33^ To obtain the apparent rate constants (*k*_app_) for an irreversible bimolecular diffusion-controlled reaction in solvents at 298.15 K, the Collins–Kimball theory^43^ and the literature were consulted to determine the steady-state Smoluchowski rate constant (*k*_D_).^33,44^2
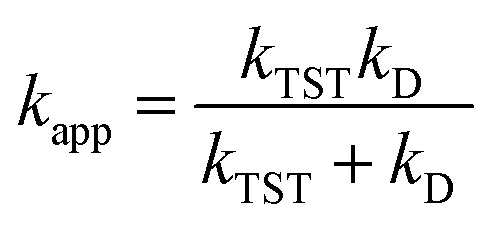

3 *k*_D_ = 4π*R*_AB_*D*_AB_*N*_A_

*D*_AB_ = *D*_A_ + *D*_B_, where *D*_A_ or *D*_B_ is the mutual diffusion coefficient of A and B as calculated using the Stokes–Einstein formulation (4).^43,45–47^4
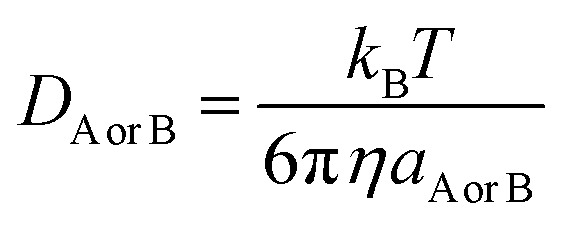


*η* is the viscosity of the solvents and *a* is the radius of the solute that was obtained in Gaussian calculations. The viscosity of isopropanol is 20.4 × 10^−4^ Pa s and that of toluene is 5.60 × 10^−4^ Pa s. Identifiable transition states had a single imaginary frequency. To ensure that each transition state is accurately associated with the pre- and post-complexes, calculations with intrinsic coordinates were conducted.

All computations for this investigation were performed utilizing the M06-2X/6-311++G(d,p) method from the Gaussian 16 software package.^48^ This model chemistry yields the most precise thermodynamics and kinetics outcomes.^49–53^ It is frequently used to assess the radical reactions with small errors in comparison to experimental data (*k*_calc_/*k*_exp_ ratio = 0.2–2.9).^16,32,33,38,54-56^ The SMD method was used to model the effects of isopropanol and toluene.^57^ AIM2000 software was used to conduct atom-in-molecule (AIM) analysis at the M06-2X/6-311++G(d,p) level.^58,59^

## 3. Results and discussion

### 3.1 Initiation reactions of TBO˙/CMO˙ in the organic solvents

**Fig. 2:**
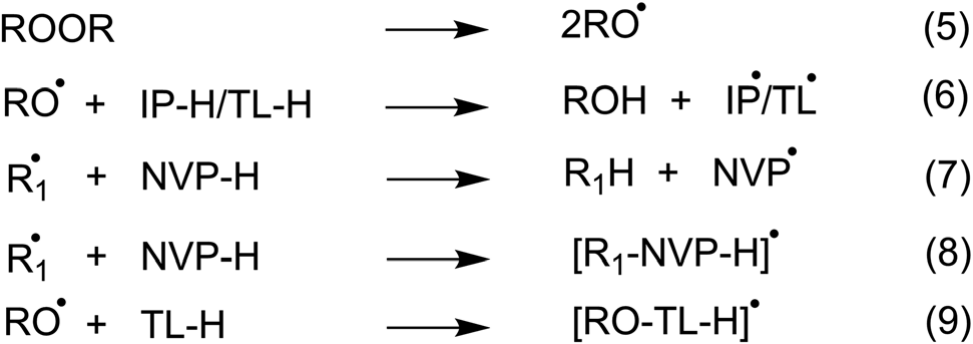
The initiation reaction of the TBO˙/CMO˙ in isopropanol (IP) and toluene (TL) (R:TB, CM; R_1_˙:TBO˙, CMO˙, IP˙, TL˙).

**Fig. 3:**
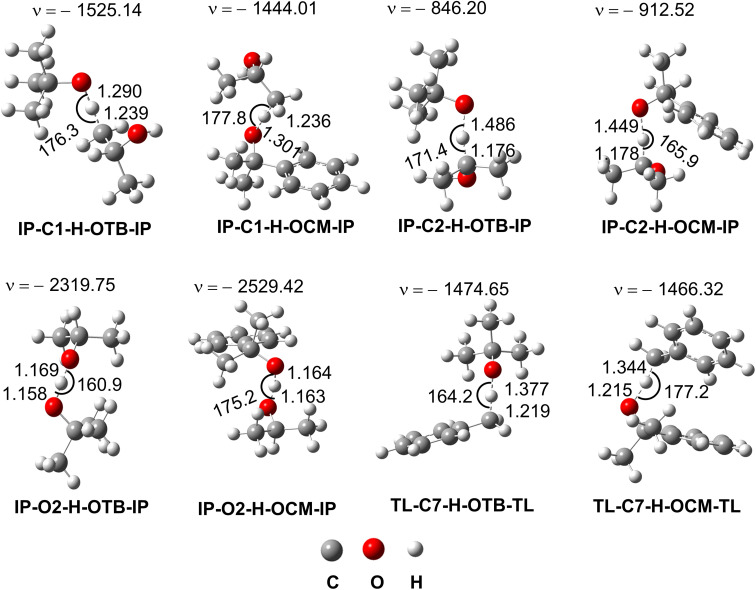
The transition states of the reactions.

To investigate the initiation reaction of the polymerization utilizing organic peroxides such as di-*tert*-butyl peroxide ((TBO)_2_) or dicumyl peroxide ((CMO)_2_) in organic solvents such as IP, or TL, all potential radical reactions could occur following the reactions 5–9 (Fig. 2). The alkoxy radicals are formed by heating peroxides in accordance with reaction 5, while the TBO˙, CMO˙ radicals can react with solvents or NVP at possible reactions, *i.e.* the formal hydrogen transfer (FHT, reactions 6, 7, and Table 1) and the radical adduct formation (RAF, reaction 8, 9 and Table 1). As shown in Table 1, the FHT mechanism for the IP/TL + TBO˙/CMO˙ reactions is thermodynamically spontaneous (Δ*G*° = −14.0 to −0.8 kcal mol^−1^), whereas the RAF mechanism is thermodynamically nonspontaneous (Δ*G*° > 0) for all of the studied solvents and radicals. Thus the kinetics of the IP/TL + TBO˙/CMO˙ reactions were computed and presented in Table 2 and Fig. 3.

**Table 1:** The calculated values of the Δ*G*° for the IP/TL + TBO˙/CMO˙ reactions in the IP and TL

Solvents	Mechanisms	Positions	TBO˙	CMO˙
IP	FHT	C1–H	−5.2	−5.4
		C2–H	−13.8	−14.0
		O2–H	−0.8	−1.0
TL	FHT	C7–H	−13.0	−13.4
	RAF	C1	11.3	12.6
		C2	10.9	12.2
		C3	9.4	10.5
		C4	8.5	9.4

**Table 2:** Calculated Δ*G*^≠^ (*k*cal mol^−1^), tunneling corrections (*κ*), rate constants (*k*_app_, *k*_r_, and *k*_overall_ M^−1^ s^−1^) and branching ratios (*Γ*,%) for the IP/TL + TBO˙/CMO˙ reactions^a^

Solvents	Mechanisms		TBO˙				CMO˙			
			Δ*G*^≠^	*κ*	*k*_app_	*Γ*	Δ*G*^≠^	*κ*	*k*_app_	*Γ*
IP	FHT	C1–H	16.7	13.0	2.70 × 10^2^	1.2	15.2	8.4	2.10 × 10^3^	2.7
		C2–H	11.9	2.0	2.30 × 10^4^	98.5	10.9	1.3	7.60 × 10^4^	96.8
		O2–H	18.3	361.9	8.00 × 10^1^	0.3	16.7	107.9	3.90 × 10^2^	0.5
	*k*_overall_				2.34 × 10^4^				7.85 × 10^4^	
TL	FHT	C7–H	14.7	8.0	8.90 × 10^2^		16.4	7.5	4.60 × 10^1^	

a *k*_overall_ = ∑*k*_app_; *Γ* = *k*_app_ × 100/*k*_overall_

As shown in Table 2, the H-abstraction at the C2–H bond defined the IP + TBO˙/CMO˙ reactions with the *k*_app_ = 2.30 × 10^4^ (*Γ* = 98.5%) and 7.60 × 10^4^ (*Γ* = 96.8%) M^−1^ s^−1^ for the TBO˙ and CMO˙ radicals, respectively, whereas the FHT reaction of the O2–H bond contributed only 0.3–0.5% in the overall rate constant. Thus the IP-C2˙, which was formed by reaction 6 in the IP solvent, is the main radical for the following reactions (*i.e.* 7 and 8). At the same time, the H-abstraction at the C7–H characterized the TL + TBO˙/CMO˙ reactions and formed the TL-C7˙ radical with *k*_app_ = 8.90 × 10^2^ and 4.60 × 10^1^ M^−1^ s^−1^ for the TBO˙ and CMO˙ radicals, respectively, however, these are slower than the IP + TBO˙/CMO˙ reactions. Thus in the IP solution, the NVP can react with three main radicals including IP-C2˙, TBO˙ and CMO˙, whereas in the TL solvent, the IP-C2˙ is replaced by the TL-C7˙ radical.

To gain insight into the structure of the TSs, the AIM analysis was used to measure the intermolecular contacts (Table S1, Fig. S2, ESI†). It was found that the TS-IP-C2-H-OTB/OCM-IP are stabilized by intermolecular contacts at the H5⋯C2, H5⋯O13, (TBO)H3⋯O2, (TBO)H3⋯H1, (TBO)H1⋯C1, (CMO)H1⋯O2, (CMO)C4⋯H1 and ring critical points (RCPs) at RCP1, RCP2 and RCP3, whereas those of the C1–H are only defined by intermolecular contacts H7⋯C1, H7⋯O13, (CMO)C6⋯H1 and RCP1, RCP2. Intermolecular contacts and RCPs define the stability of TSs of the TS-IP-O2-H-OTB/OCM-IP; however, the *E*_H–B_(O2–H12) values (*E*_H–B_(O2–H12) = −87.5 and −90.3 kcal mol^−1^) are significantly lower than the *E*_H–B_(C2–H5) values (*E*_H–B_(C2–H5) = −69.3 and −69.9 kcal mol^−1^). Therefore, the O2–H12 bond may be more difficult to break than the C2–H5 bond when forming the products. This may account for the high stability of TS-IP-C2-H-OTB/OCM-IP and the rapid H-abstraction at the C2–H bond. At the TSs of TL-C7-H + OTB/OCM reactions, the O16–H13 bond at the TS-TL-C7-H-OTB is more stable than that at the TS-TL-C7-H-OCM, which can lead to rapid H-abstraction by the TBO radical.

**Fig. 4:**
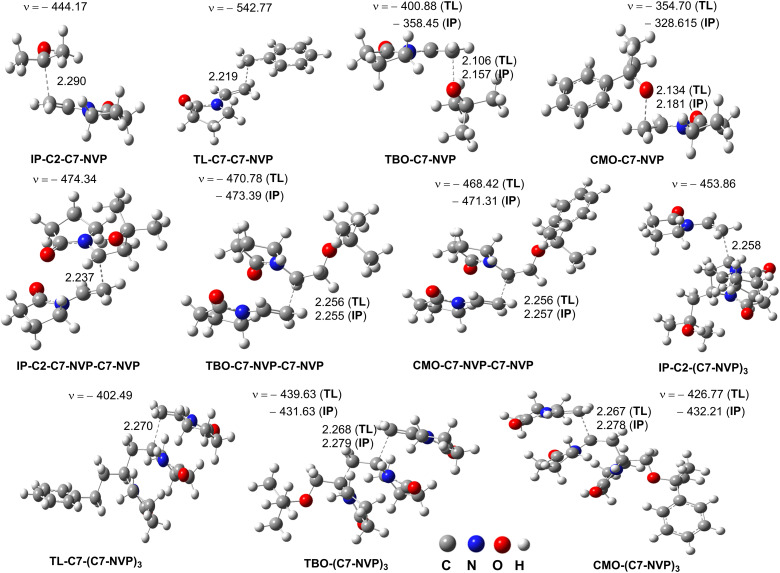
Selected transition states of the reactions.

The reaction of NVP with alkyl radicals *i.e.*IP-C2˙/TL-C7˙ was first evaluated and the results are presented in Table 3 and Fig. 4. It was found that the RAF reaction at C7 position dominated the NVP + IP-C2˙/TL-C7˙ reactions (*Γ* = 99.9%), however the rate constant of the NVP + IP-C2˙ reaction (*k*_overall_ = 6.21 × 10^3^ M^−1^ s^−1^) was about 10^3^ times faster than that of the NVP + TL-C7˙ (*k*_overall_ = 4.50 M^−1^ s^−1^). The other reactions had no contributions to the overall rate constant of the alkyl radical scavenging activity of NVP. Thus for the alkoxy radicals *i.e.*TBO˙ and CMO˙, the NVP + TBO˙/CMO˙ reactions were only focused on the RAF pathway at the C7 position (Table 3 and Fig. 4).

**Table 3:** Calculated Δ*G*^≠^ (kcal mol^−1^), tunneling corrections (*κ*), rate constants (*k*_app_, *k*_r_, and *k*_overall_ M^−1^ s^−1^) and branching ratios (*Γ*,%) for the NVP + IP-C2˙/TL-C7˙/TBO˙/CMO˙ reactions in the organic solvents

RAD/Sol.	Mechanism		Δ*G*^≠^	*κ*	*k*_app_	*Γ*
IP-C2˙/IP	FHT	C3–H	17.7	8.1	5.70	0.1
		C4–H	23.2	22.0	1.40 × 10^−3^	0.0
		C5–H	20.8	21.0	7.00 × 10^−2^	0.0
	RAF	C6	35.2	1.0	1.10 × 10^−13^	0.0
		C7	12.4	1.2	6.20 × 10^3^	99.9
	*k*_overall_				6.21 × 10^3^	
TL-C7˙/TL	FHT	C3–H	24.6	16.0	9.10 × 10^−5^	0.0
		C4–H	26.2	21.0	8.80 × 10^−6^	0.0
		C5–H	22.4	18.0	4.10 × 10^−3^	0.1
	RAF	C6	22.2	1.7	6.20 × 10^−4^	0.0
		C7	16.7	1.4	4.50	99.9
	*k*_overall_				4.50	
TBO˙/IP	RAF	C7	11.4	1.1	3.10 × 10^4^	
CMO˙/IP			9.7	1.0	5.20 × 10^5^	
TBO˙/TL			12.7	1.2	3.40 × 10^3^	
CMO˙/TL			10.5	1.1	1.50 × 10^5^	

As shown in Table 3, the alkoxy radical reactions of NVP in the IP solvent were faster than in the TL solution. The NVP + TBO˙/CMO˙ reactions in the IP solution were about 9.1 and 3.5 times faster than those in the TL solution for TBO˙ and CMO˙ respectively. It is important to notice that in the IP solution, the formed radical from the solvent (IP-C2˙) can react with NVP as fairly fast as the NVP + TBO˙/CMO˙ reactions (*k* = 10^3^–10^5^ M^−1^ s^−1^), thus the IP-C2˙ may also contribute to the propagation reactions. However, in the TL solution, the NVP + TBO˙/CMO˙ reactions were about 10^3^−10^4^ times faster than the NVP + TL-C7˙ reaction.

The AIM analysis (Table S1, Fig. S2, ESI†) indicated that energies (*E*_H–B_) of the C7⋯C/O intermolecular contacts of RAF transition states are in the range of −13.1 to −9.8 kcal mol^−1^. The replacement of the methyl group at TBO˙ by phenyl at CMO˙ could reduce the *E*_H–B_(C7⋯O) values, particularly in TL solvent. That may be a reason for the high rate constant of the CMO˙ + NVP reaction.

### 3.2 The propagation reaction

**Fig. 5:**
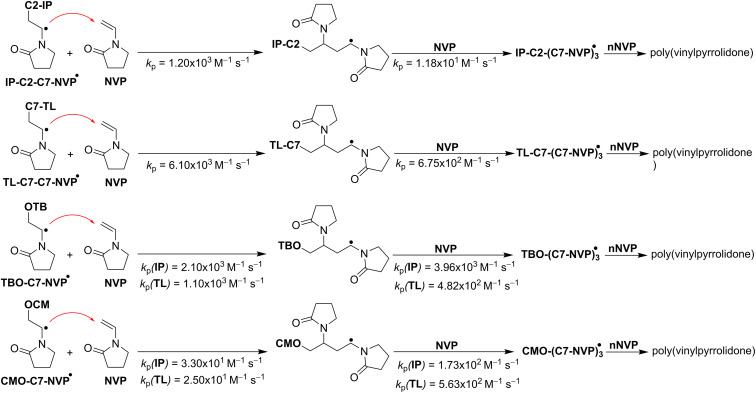
The propagation reactions.

As previously mentioned, the main intermediates of the radical process were IP-C2-C7-NVP, TL-C7-C7-NVP, TBO-C7-NVP, and CMO-C7-NVP (Table 3 and Fig. 3). These radicals were thought to follow the RAF pathway, the basic reaction mechanism of radical chain polymerization, and react with NVP at the most active site (C7). Thus, using the QM-ORSA approach,^33^ the propagation rate constant (*k*_p_) of the reaction between IP-C2-C7-NVP/TL-C7-C7-NVP/TBO-C7-NVP/CMO-C7-NVP and NVP was determined. The results are provided in Table 4, and the TSs are illustrated in Fig. 5.

**Table 4:** Calculated Δ*G*^≠^ (kcal mol^−1^), tunneling corrections (*κ*), rate constants (*k*_p_, M^−1^ s^−1^) the propagation reaction

Reactions	Solvents	Δ*G*^≠^	*κ*	*k*_p_
IP-C2-C7-NVP˙ + NVP	IP	13.4	1.2	1.20 × 10^3^
TL-C7-C7-NVP˙ + NVP	TL	12.4	1.2	6.10 × 10^3^
TBO-C7-NVP˙ + NVP	IP	13.0	1.2	2.10 × 10^3^
CMO-C7-NVP˙ + NVP		15.5	1.2	3.30 × 10^1^
TBO-C7-NVP˙ + NVP	TL	13.5	1.2	1.10 × 10^3^
CMO-C7-NVP˙ + NVP		15.7	1.2	2.50 × 10^1^
IP-C2-C7-NVP-C7-NVP˙ + NVP	IP	16.1	1.2	1.18 × 10^1^
TL-C7-C7-NVP-C7-NVP˙ + NVP	TL	13.7	1.2	6.75 × 10^2^
TBO-C7-NVP-C7-NVP˙ + NVP	IP	12.6	1.1	3.96 × 10^3^
CMO-C7-NVP-C7-NVP˙ + NVP		14.4	1.0	1.73 × 10^2^
TBO-C7-MVP-C7-NVP˙ + NVP	TL	13.9	1.2	4.82 × 10^2^
CMO-C7-NVP-C7-NVP˙ + NVP		13.7	1.0	5.63 × 10^2^

As shown in Tables 4, in the IP solution, the *k*_p_ value of the IP-C2-C7-NVP + NVP reaction (*k*_p_ = 1.20 × 10^3^ M^−1^ s^−1^) was similar to that of the TBO-C7-NVP + NVP reaction (*k*_p_ = 2.10 × 10^3^ M^−1^ s^−1^), whereas these values were about 36.4 and 63.6 (for IP-C2-C7-NVP and TBO-C7-NVP, respectively) times higher than that of the CMO-C7-NVP + NVP reaction (*k*_p_ = 3.30 × 10^1^ M^−1^ s^−1^). Therefore, the radical polymerization of NVP in the isopropanol solution with (CMO)_2_ as an initiator could produce PVP with a solvent molecule (IP-C2). That is in good agreement with the experimental data.^4^ However, when (TBO)_2_ is used as an initiator, the PVP may contain both the solvent (IP) and initiator (TB) structures.

A similar trend was also observed in the TL solvent, the TL-C7-C7-NVP/TBO-C7-NVP + NVP reactions were about 24.4 (*k*_p_ = 6.10 × 10^3^ M^−1^ s^−1^) and 4.4 (*k*_p_ = 1.10 × 10^3^ M^−1^ s^−1^) times faster than the CMO-C7-NVP + NVP reaction (*k*_p_ = 2.50 × 10^1^ M^−1^ s^−1^), respectively. Being the solvent, the amount of TL is significantly greater than that of the initiators, *i.e.* (TBO)_2_ or (CMO)_2_, thus the PVP produced this way could contain residues of both the solvent (TL) and initiators (TB, CM), despite the rate constant of the TL-C7˙ + NVP reaction being lower than those of the TBO˙/CMO˙ + NVP reactions (Table 3).

The investigation of the chain extension (adding the second NVP molecule) revealed that the *k*_p_ values (*k*_p_ = 10^1^–10^2^ M^−1^ s^−1^, Table 4, Fig. 3 and 5) were comparable to those of the initial propagation reactions. It appears that the rate constants of the propagation reactions are in the range of 10^1^–10^3^ M^−1^ s^−1^, depending on the performed radicals and solvents.

Since the atoms adjacent to the center of the radicals are identical, the propagation rate constants of CMO-C7-NVP + NVP in both IP and TL were found to be lower than those of other propagation reactions. However, the *k*_p_ values for the CMO-C7-NVP-C7-NVP + NVP were fairly similar to those of the TBO-C7-NVP-C7-NVP + NVP reactions. This could be due to the steric effects of CMO in the CMO-C7-NVP + NVP reaction.^60^

## 4. Conclusion

Using computational chemistry, the mechanism and kinetics of the alkoxy radical polymerization of *N*-vinylpyrrolidone in organic solvents, namely isopropanol, and toluene, have been successfully determined. It was discovered that both solvents, *i.e.* isopropanol and toluene could contribute to the initiation reactions alongside the initiator radicals TBO˙ and CMO˙. The rate constant varied as a function of the initiators and solvents used. The values of the constant rate of propagation were approximately 10^1^–10^3^ M^−1^ s^−1^. In all of the analyzed organic solvents, the radical polymerization of NVP with (CMO)_2_ as an initiator occurred fairly similarly with (TBO)_2_, whereas the solvents could contribute to the radical polymerization of NVP.

## Author contributions

Quan V. Vo: conceptualization, methodology, investigation, formal analysis, funding acquisition, writing – original draft, supervision. Truong Le Bich Tram, Hoang Phuoc Loc, Nguyen Thi Hoa: formal analysis, data curation, investigation, visualization, writing – original draft. Adam Mechler: project supervision, administration, software, writing – review & editing.

## Conflicts of interest

There are no conflicts to declare.

## Supplementary Material

RA-013-D3RA03820C-s001
