# Supplementary material for: The alkoxy radical polymerization of N-vinylpyrrolidone in organic solvents: theoretical insight into the mechanism and kinetics
Source: RSC Adv. 2023 Aug 4;13(34):23402–8. doi: 10.1039/d3ra03820c (PMC10401519; doi:10.1039/d3ra03820c)
Supplement: RA-013-D3RA03820C-s001 [file RA-013-D3RA03820C-s001.pdf]

Electronic Supplementary Material (ESI) for RSC Advances.
This journal is © The Royal Society of Chemistry 2023

Supporting Information (SI)

The alkoxy radical polymerization of N-vinylpyrrolidone in organic solvents: Theoretical insight into the mechanism and kinetics

Quan V. Vo[1*], Truong Le Bich Tram,[2*] Hoang Phuoc Loc[3*], Nguyen Thi Hoa[2] and Adam Mechler[4]

[1]*The University of Danang - University of Technology and Education, Danang 550000, Vietnam.*
[2]*Department of Science-Technology and Environment, The University of Danang, Danang 550000, Vietnam*
[3]*Quang Tri Teacher Training College, Dong Ha, Quang Tri, 520000, Viet Nam*
[4]*Department of Biochemistry and Chemistry, La Trobe University, Victoria 3086, Australia.*
**Corresponding authors: vvquan@ute.udn.vn;* tlbtram@ac.udn.vn; loc_hp@qtttc.edu.vn

Table of Contents

Figure S1: The IRC plots for the selected transition states in IP and TL (NVP: N-vinylpyrrolidone; FHT: formal hydrogen transfer and RAF: radical adduct formation: IP: isopropanol; TL: toluene). S2

Figure S2: Atom in molecule topological shapes of the selected TSs. Small red and yellow spheres indicate bond critical points (BCPs) and ring critical points (RCPs), respectively. S3

Table S1: Selected parameters at the BCPs and bond critical points (BCPs) and ring critical points (RCPs), for the transition states at 298.15 K...S4

Table S2: The Cartesian coordinates and energies of transition states following the RAF and FHT mechanisms in IP and TL (NVP: N-vinylpyrrolidone; FHT: formal hydrogen transfer and RAF: radical adduct formation: IP: isopropanol; TL: toluene)...S7

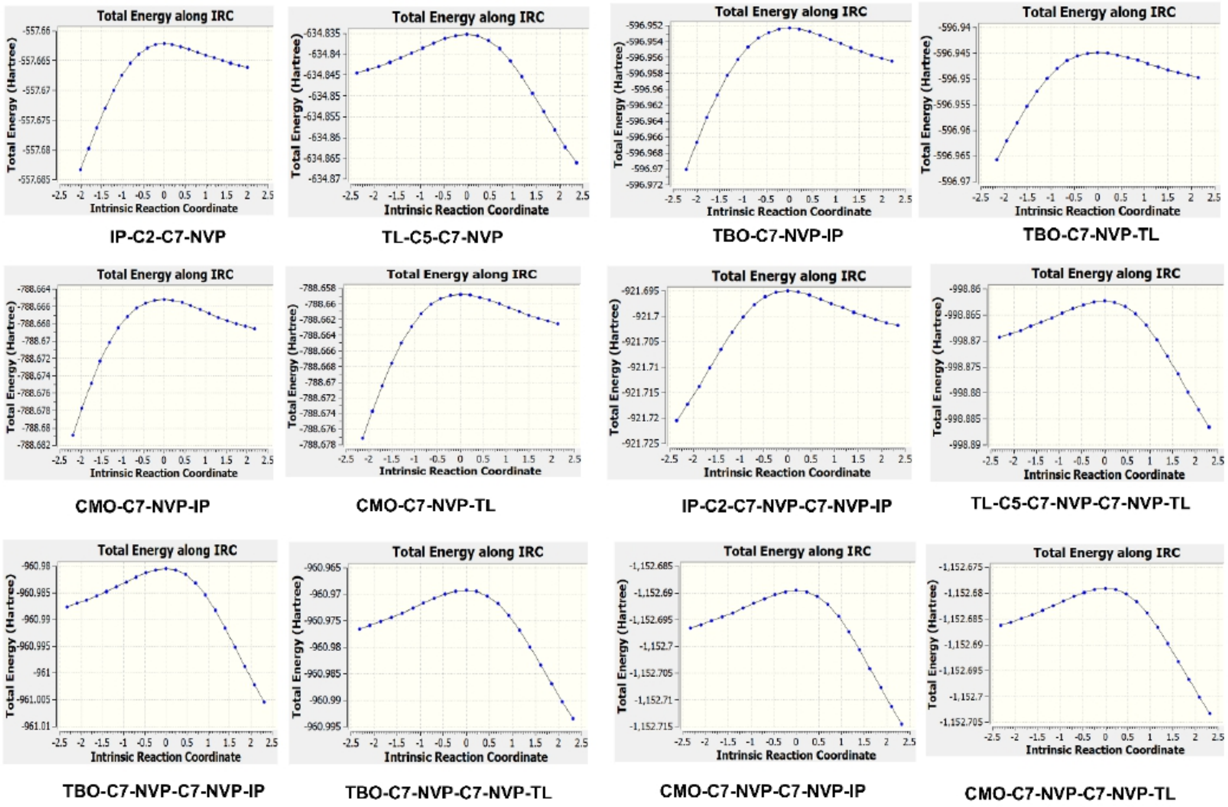

Figure S1: The IRC plots for the selected transition states in IP and TL (NVP: N-vinylpyrrolidone; FHT: formal hydrogen transfer and RAF: radical adduct formation: IP: isopropanol; TL: toluene).

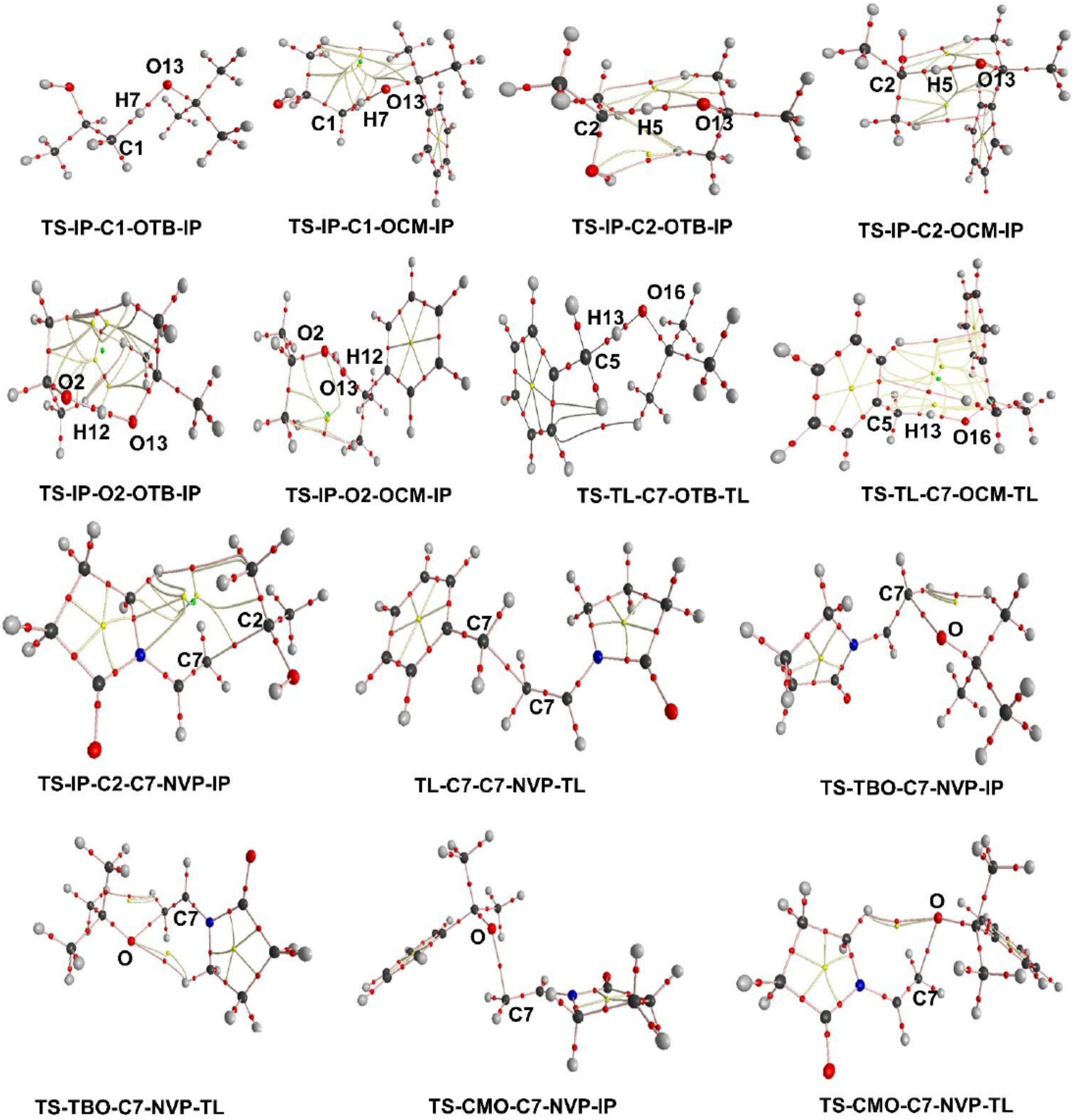

Figure S2: **Atom in molecule topological shapes of the selected TSs. Small red and yellow spheres indicate bond critical points (BCPs) and ring critical points (RCPs), respectively.**

Table S1: Selected parameters at the BCPs and bond critical points (BCPs) and ring critical points (RCPs), for the transition states at 298.15 K

Contacts	ρ(r) (au)	∇^2ρ(r) (au)	G(r)[a] (au)	V(r)[b] (au)	G(r)/|V(r)|	H(r)[c] (au)	E_{HB}[d] (kcal/.mol)
**IP-C1-H-OTB-IP**							
C1···H7	0.1801	-0.3716	0.0393	-0.1715	0.2291	-0.1322	-53.8
O13···H7	0.1405	0.0366	0.0883	-0.1675	0.5273	-0.0792	-52.5
**IP-C1-H-OCM-IP**							
C1···H7	0.1812	-0.3782	0.0393	-0.1731	0.2269	-0.1339	-54.3
O13···H7	0.1368	0.0478	0.0866	-0.1613	0.5371	-0.0747	-50.6
(CMO)C6···H1	0.0043	0.0145	0.0029	-0.0021	1.3465	0.0007	-0.7
RCP1	0.0038	0.0142	0.0029	-0.0022	1.3206	0.0007	-0.7
RCP2	0.0038	0.0138	0.0028	-0.0021	1.3201	0.0007	-0.7
**IP-C2-H-OTB-IP**							
C2···H5	0.2218	-0.5999	0.0354	-0.2208	0.1603	-0.1854	-69.3
O13···H5	0.0938	0.1389	0.0675	-0.1002	0.6733	-0.0327	-31.4
(TBO)H3···O2	0.0058	0.0211	0.0045	-0.0036	1.2276	0.0008	-1.1
(TBO)H3···H1	0.0058	0.0175	0.0037	-0.0031	1.2102	0.0006	-1.0
(TBO)H1···C1	0.0053	0.0177	0.0036	-0.0028	1.2858	0.0008	-0.9
RCP1	0.0058	0.0220	0.0046	-0.0037	1.2362	0.0009	-1.2
RCP2	0.0055	0.0193	0.0040	-0.0032	1.2585	0.0008	-1.0
RCP3	0.0046	0.0170	0.0035	-0.0027	1.2853	0.0008	-0.8
**IP-C2-H-OCM-IP**							
C2···H5	0.2234	-0.6099	0.0352	-0.2229	0.1579	-0.1877	-69.9
O13···H5	0.0894	0.1399	0.0643	-0.0937	0.6867	-0.0293	-29.4
(CMO)H1···O2	0.0079	0.0245	0.0055	-0.0049	1.1245	0.0006	-1.5
(CMO)C4···H1	0.0070	0.0215	0.0045	-0.0037	1.2249	0.0008	-1.2
RCP1	0.0059	0.0233	0.0050	-0.0041	1.2141	0.0009	-1.3
RCP2	0.0059	0.0208	0.0043	-0.0034	1.2617	0.0009	-1.1
**IP-O2-H-OTB-IP**							
O2···H12	0.1871	-0.3096	0.1008	-0.2790	0.3613	-0.1782	-87.5
O13···H12	0.1868	-0.3073	0.1007	-0.2782	0.3620	-0.1775	-87.3
(TBO)H1···H1	0.0044	0.0135	0.0028	-0.0022	1.2530	0.0006	-0.7
(TBO)H3···H1	0.0072	0.0194	0.0042	-0.0036	1.1803	0.0006	-1.1

(TBO)H3···H3	0.0052	0.0154	0.0032	-0.0027	1.2245	0.0006	-0.8
RCP1	0.0037	0.0123	0.0025	-0.0020	1.2836	0.0006	-0.6
RCP2	0.0039	0.0133	0.0027	-0.0021	1.2751	0.0006	-0.7
RCP3	0.0046	0.0162	0.0034	-0.0027	1.2627	0.0007	-0.8
RCP4	0.0042	0.0148	0.0030	-0.0023	1.2879	0.0007	-0.7
XANH	0.0030	0.0117	0.0023	-0.0017	1.3497	0.0006	-0.5
**IP-O2-H-OCM-IP**							
O2···H12	0.1917	-0.3371	0.1018	-0.2879	0.3536	-0.1861	-90.3
O13···H12	0.1861	-0.2844	0.1015	-0.2741	0.3703	-0.1726	-86.0
(CMO)C1···H1	0.0048	0.0162	0.0032	-0.0024	1.3431	0.0008	-0.8
RCP1	0.0043	0.0156	0.0032	-0.0024	1.3013	0.0007	-0.8
RCP2	0.0042	0.0157	0.0032	-0.0024	1.3135	0.0008	-0.8
**TL-C5-H-OTB-TL**							
C5···H13	0.1921	-0.4313	0.0384	-0.1846	0.2079	-0.1462	-57.9
O16···H13	0.1209	0.0861	0.0801	-0.1386	0.5776	-0.0585	-43.5
C2···H1	0.0048	0.0150	0.0031	-0.0024	1.2734	0.0007	-0.8
**TL-C5-H-OCM-TL**							
C5···H13	0.1911	-0.4355	0.0361	-0.1811	0.1995	-0.1450	-56.8
O16···H13	0.1109	0.1032	0.0745	-0.1231	0.6047	-0.0487	-38.6
H2···C2(CMO)	0.0058	0.0154	0.0033	-0.0027	1.2205	0.0006	-0.8
HC2···H5(CMO)	0.0053	0.0139	0.0030	-0.0025	1.2054	0.0005	-0.8
RCP1	0.0033	0.0097	0.0020	-0.0016	1.2549	0.0004	-0.5
RCP2	0.0032	0.0102	0.0021	-0.0017	1.2640	0.0004	-0.5
RCP3	0.0044	0.0149	0.0031	-0.0024	1.2682	0.0006	-0.8
**IP-C2-C7-NVP-IP**							
C2···C7	0.0493	0.0498	0.0218	-0.0311	0.7003	-0.0093	-9.8
(IP)C1···H5	0.0064	0.0209	0.0043	-0.0034	1.2719	0.0009	-1.1
RCP1	0.0053	0.0202	0.0042	-0.0034	1.2351	0.0008	-1.1
RCP2	0.0049	0.0179	0.0037	-0.0029	1.2617	0.0008	-0.9
**TL-C7-C7-NVP-TL**							
C5···C7	0.0494	0.1291	0.0342	-0.0362	0.9454	-0.0020	-11.4
(TBO)H1···H7	0.0079	0.0263	0.0054	-0.0043	1.2651	0.0011	-1.4
RCP	0.0076	0.0300	0.0061	-0.0048	1.2848	0.0014	-1.5
**TBO-C7-NVP-IP**							
O···C7	0.0473	0.1246	0.0327	-0.0343	0.9545	-0.0016	-10.7
**TBO-C7-NVP-TL**							

O···C7	0.0550	0.1397	0.0383	-0.0416	0.9193	-0.0034	-13.1
(TBO)H1···H7	0.0077	0.0256	0.0053	-0.0042	1.2659	0.0011	-1.3
O···H5	0.0079	0.0281	0.0062	-0.0053	1.1637	0.0009	-1.7
RCP1	0.0075	0.0289	0.0059	-0.0046	1.2869	0.0013	-1.4
RCP2	0.0076	0.0305	0.0065	-0.0054	1.2039	0.0011	-1.7
**CMO-C7-NVP-IP**							
O···C7	0.0473	0.1246	0.0327	-0.0343	0.9545	-0.0016	-10.7
**CMO-C7-NVP-TL**							
O···C7	0.0522	0.1343	0.0362	-0.0389	0.9312	-0.0027	-12.2
O···H5	0.0070	0.0259	0.0056	-0.0048	1.1756	0.0008	-1.5
RCP	0.0070	0.0268	0.0058	-0.0049	1.1895	0.0009	-1.5
a) electron kinetic energy density; b) electron potential energy density; c) total electron energy density; d) individual energies of each hydrogen bond.							

Table S2: The Cartesian coordinates and energies of transition states following the RAF and FHT mechanisms in IP and TL (NVP: N-vinylpyrrolidone; FHT: formal hydrogen transfer and RAF: radical adduct formation: IP: isopropanol; TL: toluene).

**Name**	**IP-C1-H-OTB-FHT-IP**
Cartesian Coordinates	Frequency and Energy
O 1.78846600 -0.01712300 0.07461100<br>C 0.36148200 -0.01131600 0.04733000<br>C -0.18632100 1.22707600 0.71442900<br>C -0.15569500 -0.13747900 -1.38238100<br>H 0.06574800 -0.89511300 0.61875200<br>H -1.27205900 1.23306000 0.81366500<br>H 0.27066900 1.24283700 1.86549500<br>H 0.18781500 2.15799700 0.27928900<br>H -1.24585000 -0.20485100 -1.39488900<br>H 0.25598000 -1.03512100 -1.84971300<br>H 0.14425800 0.73489800 -1.97113800<br>H 2.10242000 0.74460000 -0.42827400<br>O 0.77786100 1.18423400 3.05034700<br>C -0.26678100 0.87550900 3.95878300<br>C -1.29792600 2.00101800 3.99531200<br>C 0.45276600 0.76900600 5.31048000<br>C -0.91583800 -0.45967800 3.60048700<br>H -1.78646700 2.10360100 3.02243900<br>H -0.81453700 2.94916000 4.24395600<br>H -2.06668500 1.79406400 4.74427000<br>H 0.94934300 1.71066700 5.55470800<br>H -0.27884600 0.54497500 6.09089500<br>H 1.19726000 -0.02968800 5.28560400<br>H -1.44042600 -0.38837600 2.64366700<br>H -1.64138200 -0.74867500 4.36520900<br>H -0.15607200 -1.24197200 3.52657200	Zero-point correction= 0.227462 (Hartree/Particle)<br>Thermal correction to Energy= 0.239972<br>Thermal correction to Enthalpy= 0.240916<br>Thermal correction to Gibbs Free Energy= 0.188553<br>Sum of electronic and zero-point Energies= -427.054162<br>Sum of electronic and thermal Energies= -427.041652<br>Sum of electronic and thermal Enthalpies= -427.040708<br>Sum of electronic and thermal Free Energies= -427.093071
**Name**	**IP-C2-H-OTB- FHT-IP**
Cartesian Coordinates	Frequency and Energy
O 0.80417800 1.63541300 -0.06721700<br>C 0.60167000 0.24914300 -0.19303800<br>C -0.42990600 -0.28411300 0.77911800<br>C 0.31895500 -0.06324800 -1.64304200<br>H 1.63241900 -0.27816600 0.02851700<br>H -0.51848900 -1.36824800 0.68525300<br>H -0.16296600 -0.04329900 1.81162300<br>H -1.40375900 0.16668100 0.55864100<br>H 0.24008500 -1.14216900 -1.78854000<br>H 1.11969200 0.31969800 -2.27960800<br>H -0.62432800 0.40225900 -1.94728000<br>H 0.89851900 1.85779400 0.86740900<br>O 3.00135500 -0.75366600 0.00867300<br>C 3.39093600 -0.96463800 1.34558800<br>C 3.15262600 0.29162300 2.18367000<br>C 4.89905700 -1.25118300 1.24179800<br>C 2.65924300 -2.16765700 1.93679100<br>H 2.07995300 0.47026400 2.30524800<br>H 3.60474200 1.16211200 1.70163900<br>H 3.58925000 0.17451100 3.17873700<br>H 5.42417100 -0.38836500 0.82677000	Zero-point correction= 0.228662 (Hartree/Particle)<br>Thermal correction to Energy= 0.241077<br>Thermal correction to Enthalpy= 0.242022<br>Thermal correction to Gibbs Free Energy= 0.190625<br>Sum of electronic and zero-point Energies= -427.062714<br>Sum of electronic and thermal Energies= -427.050298<br>Sum of electronic and thermal Enthalpies= -427.049354<br>Sum of electronic and thermal Free Energies= -427.100751

H	5.29085000	-1.45841100	2.24087400	
H	5.08067400	-2.11989900	0.60537900	
H	1.58196500	-1.98049400	1.95678700	
H	2.99323200	-2.36052000	2.95965700	
H	2.84704900	-3.05926400	1.33323700	
**Name**				**IP-O2-H-OTB- FHT-IP**
Cartesian Coordinates				Frequency and Energy
O	0.62270300	1.41526000	-1.42820300	Zero-point correction= 0.226163 (Hartree/Particle)
C	0.88807800	0.31197900	-0.60070200	Thermal correction to Energy= 0.238544
C	1.59253100	0.71907600	0.68392900	Thermal correction to Enthalpy= 0.239489
C	-0.35220500	-0.53937600	-0.35927500	Thermal correction to Gibbs Free Energy= 0.187579
H	1.59032600	-0.28396000	-1.21080200	Sum of electronic and zero-point Energies= -427.051905
H	1.92170600	-0.16691300	1.23251900	Sum of electronic and thermal Energies= -427.039523
H	2.46695600	1.33428000	0.46091400	Sum of electronic and thermal Enthalpies= -427.038579
H	0.91818100	1.28934900	1.32846300	Sum of electronic and thermal Free Energies= -427.090489
H	-0.07564900	-1.46307900	0.15306400	
H	-0.83829500	-0.79235100	-1.30378700	
H	-1.06214700	0.00848400	0.26723900	
H	-0.43741800	1.88394200	-1.33297100	
O	-1.49142600	2.33746600	-1.14072200	
C	-1.56925500	3.35254600	-0.16347800	
C	-2.74373000	4.22735700	-0.64544100	
C	-0.28483000	4.17695200	-0.12920100	
C	-1.88980700	2.75437900	1.20258800	
H	-3.65500800	3.63095300	-0.72291100	
H	-2.51713000	4.66522000	-1.61924900	
H	-2.90554000	5.02831700	0.07989600	
H	-0.04056200	4.54659200	-1.12815400	
H	-0.40753300	5.03004800	0.54186600	
H	0.54686700	3.56640000	0.23237900	
H	-2.80666200	2.16182500	1.15063900	
H	-2.02607900	3.54517800	1.94456200	
H	-1.07505900	2.10771900	1.53645200	
**Name**				**IP-C1-H-OCM- FHT-IP**
Cartesian Coordinates				Frequency and Energy
O	3.46888700	-4.41227400	-3.39739100	Zero-point correction= 0.281251 (Hartree/Particle)
C	3.76970100	-3.50120100	-2.33947200	Thermal correction to Energy= 0.296650
C	4.01509500	-2.10059900	-2.87823800	Thermal correction to Enthalpy= 0.297595
C	2.69439400	-3.53979500	-1.28048500	Thermal correction to Gibbs Free Energy= 0.237128
H	4.69565600	-3.88518100	-1.89473300	Sum of electronic and zero-point Energies= -618.712996
H	4.29121400	-1.41964800	-2.06945100	Sum of electronic and thermal Energies= -618.697596
H	4.82285800	-2.11463800	-3.61290900	Sum of electronic and thermal Enthalpies= -618.696652
H	3.10992100	-1.71803800	-3.35964300	Sum of electronic and thermal Free Energies= -618.757119
H	2.86178600	-2.85495400	-0.44789100	
H	2.44751000	-4.54703000	-0.94057100	
H	1.65236200	-3.15615800	-1.82334200	
H	2.66197000	-4.10796900	-3.83211300	
O	0.53391200	-2.75131300	-2.35107800	
C	0.01799900	-1.75819900	-1.48673700	
C	-0.16949600	-2.32084500	-0.07604300	
C	0.89898600	-0.51041100	-1.52833000	
C	-1.36507700	-1.43987200	-2.08655900	
C	-0.04265000	-1.52213800	1.06168900	
C	-0.51302900	-3.66633600	0.08638000	

C	-0.24813100	-2.05753400	2.33263700	
C	-0.71730300	-4.20196700	1.35352500	
C	-0.58470400	-3.39823400	2.48393700	
H	1.06242000	-0.23330300	-2.57193100	
H	0.42389400	0.33313000	-1.02435700	
H	1.86791700	-0.69871400	-1.05831100	
H	-1.25137200	-1.05146800	-3.10117800	
H	-1.85824700	-0.68568400	-1.46882400	
H	-1.98506900	-2.33809900	-2.11104900	
H	0.22161100	-0.47508900	0.97112600	
H	-0.61256900	-4.29909700	-0.78850600	
H	-0.14066900	-1.42185800	3.20471700	
H	-0.97896800	-5.24923000	1.45877200	
H	-0.74112700	-3.81472300	3.47269100	
**Name**				**IP-C2-H-OCM- FHT-IP**
Cartesian Coordinates				Frequency and Energy
O	2.36234700	-2.90708600	-0.11327300	Zero-point correction= 0.282516 (Hartree/Particle)
C	1.87607400	-3.90214300	-0.97827900	Thermal correction to Energy= 0.297835
C	2.97838100	-4.74872500	-1.58136100	Thermal correction to Enthalpy= 0.298780
C	0.80873100	-4.68706600	-0.25259100	Thermal correction to Gibbs Free Energy= 0.239730
H	1.36420000	-3.38903900	-1.90441400	Sum of electronic and zero-point Energies= -618.721214
H	2.55858300	-5.46185800	-2.29398900	Sum of electronic and thermal Energies= -618.705894
H	3.70546000	-4.12310200	-2.10626900	Sum of electronic and thermal Enthalpies= -618.704950
H	3.49630000	-5.30254600	-0.79145600	Sum of electronic and thermal Free Energies= -618.764000
H	0.31001900	-5.37436000	-0.93879300	
H	0.06258000	-4.01706700	0.18091400	
H	1.26950000	-5.26913400	0.55296800	
H	3.13116000	-2.48531100	-0.51698100	
O	0.77855100	-2.92976700	-3.17060500	
C	0.06570000	-1.76200800	-2.83456500	
C	-0.95110100	-2.09051400	-1.73740700	
C	1.03250700	-0.64046600	-2.46016900	
C	-0.66968400	-1.38571800	-4.13747700	
C	-1.02395800	-1.37792600	-0.54155500	
C	-1.81443600	-3.17542000	-1.92421200	
C	-1.93839900	-1.74273000	0.44814200	
C	-2.72663300	-3.53939500	-0.94198300	
C	-2.79091800	-2.82259400	0.25268500	
H	1.74027900	-0.50314500	-3.28039600	
H	0.50548100	0.30239400	-2.29766800	
H	1.59050000	-0.89022800	-1.55542300	
H	0.05679400	-1.18203200	-4.92694800	
H	-1.26345300	-0.48584300	-3.95913900	
H	-1.33212200	-2.18997100	-4.46018900	
H	-0.36418500	-0.53792200	-0.36067700	
H	-1.75613700	-3.75279000	-2.84091500	
H	-1.97666700	-1.17976100	1.37430000	
H	-3.38477400	-4.38606000	-1.10416000	
H	-3.49900000	-3.10678400	1.02309100	
**Name**				**IP-O2-H-OCM- FHT-IP**
Cartesian Coordinates				Frequency and Energy
O	1.33483600	-3.59097000	-0.46097800	Zero-point correction= 0.280459 (Hartree/Particle)
C	2.67981600	-3.99946200	-0.36120900	Thermal correction to Energy= 0.295912
C	2.95728500	-5.24613700	-1.18679100	Thermal correction to Enthalpy= 0.296857

C	3.63956100	-2.85782000	-0.67144300	Thermal correction to Gibbs Free Energy= 0.236225
H	2.77370100	-4.24650000	0.70980200	Sum of electronic and zero-point Energies= -618.710586
H	3.96613900	-5.61491800	-0.98883100	Sum of electronic and thermal Energies= -618.695132
H	2.24328600	-6.03597100	-0.94384900	Sum of electronic and thermal Enthalpies= -618.694188
H	2.87968100	-5.01644300	-2.25361600	Sum of electronic and thermal Free Energies= -618.754819
H	4.66798600	-3.16798100	-0.47283100	
H	3.41230600	-1.98429800	-0.05738700	
H	3.55637100	-2.58228900	-1.72684600	
H	1.01054100	-3.53294600	-1.57186800	
O	0.91144900	-3.16307800	-2.67698500	
C	0.20790800	-1.93934000	-2.72008800	
C	-1.06696900	-2.03227900	-1.88203800	
C	1.12993400	-0.79129100	-2.31387300	
C	-0.17082700	-1.79347100	-4.20975400	
C	-1.53817600	-0.96388800	-1.11883200	
C	-1.80539100	-3.21964100	-1.90230600	
C	-2.72103800	-1.08086800	-0.38946800	
C	-2.98331300	-3.33850200	-1.17384300	
C	-3.44654400	-2.26643900	-0.41330900	
H	2.04723800	-0.85305700	-2.90230900	
H	0.66252100	0.17571600	-2.50837000	
H	1.38310400	-0.85722900	-1.25314500	
H	0.73342100	-1.74532900	-4.81987400	
H	-0.74186400	-0.87114500	-4.33766200	
H	-0.78163200	-2.63819400	-4.53258400	
H	-0.98757900	-0.03170200	-1.07871400	
H	-1.44973200	-4.05949900	-2.48981700	
H	-3.07032400	-0.24118300	0.20125900	
H	-3.54068900	-4.26838200	-1.19915300	
H	-4.36470600	-2.35694600	0.15616200	
**Name**				**TL-C5-H-OTB-FHT-TL**
Cartesian Coordinates				Frequency and Energy
C	-0.86735100	0.50867500	0.29678600	Zero-point correction= 0.248098 (Hartree/Particle)
C	0.37415700	1.13265400	0.33299500	Thermal correction to Energy= 0.261257
C	0.53652000	2.43575200	-0.15179400	Thermal correction to Enthalpy= 0.262201
C	-0.58025900	3.09431200	-0.68291200	Thermal correction to Gibbs Free Energy= 0.206359
C	-1.82057000	2.47137400	-0.72225400	Sum of electronic and zero-point Energies= -504.216522
C	-1.96882300	1.17560700	-0.23218900	Sum of electronic and thermal Energies= -504.203362
H	-0.97631700	-0.49830200	0.68304900	Sum of electronic and thermal Enthalpies= -504.202418
H	1.22914200	0.60998900	0.74978300	Sum of electronic and thermal Free Energies= -504.258261
H	-0.46564100	4.10207200	-1.06883000	
H	-2.67444700	2.99598800	-1.13537300	
H	-2.93712800	0.68982200	-0.26161300	
C	1.86108500	3.09523200	-0.14448200	
H	2.29913800	3.05179500	-1.27705200	
H	1.82395500	4.17026700	0.03616800	
H	2.59911400	2.60967500	0.49337700	
O	2.74190100	3.04983500	-2.54629300	
C	3.67885100	2.01549800	-2.70125300	
C	3.96095100	2.01134800	-4.21377600	
C	3.08448200	0.67190100	-2.27661100	
C	4.96109800	2.32225900	-1.92711600	
H	4.34305000	2.98302600	-4.53197800	
H	3.04774000	1.79128800	-4.76939300	

H 4.70815700 1.24622000 -4.43920900<br>H 2.12475000 0.50963000 -2.77252900<br>H 3.75945400 -0.14856500 -2.53344000<br>H 2.92011900 0.65045500 -1.19609400<br>H 5.37244200 3.28284100 -2.24506400<br>H 5.71288600 1.54586300 -2.09157400<br>H 4.75391900 2.37460000 -0.85472800	
**Name**	**TL-C5-H-OCM-FHT-TL**
Cartesian Coordinates	Frequency and Energy
C -0.13731900 -0.30794400 -1.16620100<br>C 0.87258000 0.61611400 -0.93549000<br>C 0.58261700 1.86266300 -0.36196400<br>C -0.73984800 2.14552200 -0.00305800<br>C -1.74989500 1.21542600 -0.22503900<br>C -1.45322400 -0.01012600 -0.81429500<br>H 0.10148900 -1.26557400 -1.61516500<br>H 1.89843700 0.37767700 -1.20055100<br>H -0.96941900 3.10110900 0.45814000<br>H -2.76888000 1.44619800 0.06415200<br>H -2.24086900 -0.73346800 -0.99118300<br>C 1.65645500 2.85489500 -0.13613200<br>H 2.05688400 3.30352100 -1.19748700<br>H 1.33074900 3.74915300 0.39557200<br>H 2.55133600 2.42890500 0.31692100<br>O 2.62256700 4.09225800 -2.17371400<br>C 2.62338400 3.40814400 -3.39913300<br>C 3.45415700 2.12538000 -3.29674300<br>C 1.20422400 3.15958700 -3.90410500<br>C 3.33941000 4.39287600 -4.35432600<br>C 3.03521500 0.91340900 -3.84457100<br>C 4.67036400 2.16217600 -2.60932900<br>C 3.80533100 -0.23904500 -3.69494100<br>C 5.43860800 1.01465700 -2.45494000<br>C 5.00531800 -0.19451400 -2.99526800<br>H 0.63785600 4.08726700 -3.82933300<br>H 1.20301700 2.84276200 -4.94959500<br>H 0.70493700 2.39204300 -3.30420100<br>H 2.76095300 5.31453700 -4.44273100<br>H 3.42674600 3.92589900 -5.33815600<br>H 4.33813500 4.62925300 -3.98532300<br>H 2.09274600 0.84858200 -4.37409600<br>H 5.00492300 3.10044800 -2.18127000<br>H 3.45865700 -1.17344000 -4.12167100<br>H 6.37451600 1.06103000 -1.90957100<br>H 5.60032500 -1.09204600 -2.87224800	Zero-point correction= 0.301566 (Hartree/Particle)<br>Thermal correction to Energy= 0.316879<br>Thermal correction to Enthalpy= 0.317824<br>Thermal correction to Gibbs Free Energy= 0.257384<br>Sum of electronic and zero-point Energies= -695.874096<br>Sum of electronic and thermal Energies= -695.858782<br>Sum of electronic and thermal Enthalpies= -695.857838<br>Sum of electronic and thermal Free Energies= -695.918278
**Name**	**NVP-C7-C2-IP-RAF-IP**
Cartesian Coordinates	Frequency and Energy
O -0.84732800 -1.75951600 -0.81581300<br>N 0.36422700 0.11010400 -0.26191200<br>C -1.25947300 1.37381800 0.86861700<br>C 0.13157800 1.46872700 0.23041600<br>C -1.93676500 0.23715500 0.10077400<br>C -0.78258400 -0.61662800 -0.37324000<br>C 1.59583000 -0.33382800 -0.74707500	Zero-point correction= 0.240432 (Hartree/Particle)<br>Thermal correction to Energy= 0.253933<br>Thermal correction to Enthalpy= 0.254877<br>Thermal correction to Gibbs Free Energy= 0.199301<br>Sum of electronic and zero-point Energies= -557.421791<br>Sum of electronic and thermal Energies= -557.408291<br>Sum of electronic and thermal Enthalpies= -557.407347

C	2.75777000	0.35432300	-0.60967000	Sum of electronic and thermal Free Energies=	-557.462923
H	-1.79853000	2.31773100	0.80719700		
H	-1.15713800	1.10018900	1.92069400		
H	0.16104800	2.16938200	-0.61093400		
H	0.90240300	1.75159000	0.94714900		
H	-2.46080800	0.59868800	-0.79048300		
H	-2.63711000	-0.35504300	0.68911000		
H	1.55872300	-1.32204300	-1.18861600		
H	3.63065800	-0.01345400	-1.13539900		
H	2.77224800	1.39347000	-0.29895300		
O	3.86497500	-1.66921200	1.15856600		
C	3.76546400	-0.30472300	1.33841800		
C	2.77952400	0.13210500	2.37134100		
C	5.09617500	0.35994500	1.26528900		
H	2.70356000	1.22115000	2.39527300		
H	1.78691300	-0.28464100	2.16474200		
H	3.08620200	-0.21072800	3.37033500		
H	4.97982700	1.44512300	1.22559300		
H	5.64684000	0.03149200	0.37986800		
H	5.70583400	0.11897700	2.14863500		
H	2.98029200	-2.05795300	1.18751100		
**Name**				**NVP-C7-C5-TL-RAF-IP**	
Cartesian Coordinates				Frequency and Energy	
O	-0.97835100	-1.85199400	-0.24646300	Zero-point correction=	0.261822 (Hartree/Particle)
N	0.43303900	-0.05135700	-0.02292800	Thermal correction to Energy=	0.275751
C	-1.12498600	1.68086000	0.27913200	Thermal correction to Enthalpy=	0.276695
C	0.36066900	1.40602400	0.00096400	Thermal correction to Gibbs Free Energy=	0.217375
C	-1.84070300	0.44240700	-0.26420100	Sum of electronic and zero-point Energies=	-634.573480
C	-0.79978400	-0.65595200	-0.17369000	Sum of electronic and thermal Energies=	-634.559551
C	1.61592300	-0.76684400	0.00597700	Sum of electronic and thermal Enthalpies=	-634.558607
C	2.84959400	-0.19404700	-0.09884600	Sum of electronic and thermal Free Energies=	-634.617928
H	-1.45895500	2.60951900	-0.18064900		
H	-1.28222800	1.75562500	1.35645900		
H	0.68646400	1.81283100	-0.96273200		
H	1.01296500	1.80202000	0.78109200		
H	-2.10811000	0.55173900	-1.32009700		
H	-2.73964200	0.16096900	0.28229300		
H	1.46983700	-1.83996900	0.00779300		
H	3.71766300	-0.81526000	0.08052900		
H	2.98693900	0.87045300	0.05052700		
C	5.66794600	2.97420600	-2.13860600		
C	4.50871800	2.21891200	-2.22734000		
C	4.55684400	0.80870800	-2.22263000		
C	5.82260900	0.19622100	-2.10820800		
C	6.97845000	0.95648000	-2.01906900		
C	6.91113200	2.34992600	-2.03410800		
H	5.60651700	4.05673400	-2.15303900		
H	3.54526900	2.71235400	-2.31251100		
H	5.88332800	-0.88751100	-2.09809300		
H	7.94054100	0.46287700	-1.93889400		
H	7.81608300	2.94172800	-1.96617500		
C	3.35361200	0.02547100	-2.24961400		
H	3.42636800	-1.03408000	-2.46405000		
H	2.42975900	0.50380300	-2.55457700		

**Name**	**NVP-C7-OTB-RAF-IP**
Cartesian Coordinates	Frequency and Energy
O 0.33253100 -2.04930000 0.07907200<br>N 0.58290600 0.23197800 -0.06131700<br>C -1.56517800 0.93768600 0.57471500<br>C -0.25548600 1.43463700 -0.05001400<br>C -1.60135000 -0.55120100 0.22435000<br>C -0.14722400 -0.93075300 0.08873900<br>C 1.93281900 0.23411300 -0.30364300<br>C 2.66552200 1.36239400 -0.49347100<br>H -2.42522600 1.48734300 0.19689200<br>H -1.52028400 1.06199700 1.65793100<br>H -0.39048600 1.79086100 -1.07582700<br>H 0.22376200 2.21695900 0.53785100<br>H -2.06989900 -0.73130700 -0.74923100<br>H -2.09394000 -1.18338500 0.96195300<br>H 2.38863900 -0.74850900 -0.27424100<br>H 3.70940600 1.25884000 -0.75510900<br>H 2.20058300 2.32484300 -0.65634400<br>O 2.91248100 1.94142800 1.57022900<br>C 4.13412900 1.52102900 2.11883100<br>C 4.02001800 1.90343300 3.60745300<br>C 5.31708600 2.26013800 1.48958000<br>C 4.31680000 0.00525800 2.00040400<br>H 3.19075100 1.36752500 4.07471800<br>H 3.85129300 2.97774400 3.71281700<br>H 4.94662400 1.64245900 4.12568000<br>H 5.17842700 3.34062800 1.58372100<br>H 6.25390500 1.98671500 1.98314800<br>H 5.41033300 2.01467700 0.42862900<br>H 3.43441700 -0.51308800 2.38511400<br>H 5.19075400 -0.31857200 2.57235600<br>H 4.46646200 -0.28452700 0.95715500	Zero-point correction= 0.269281 (Hartree/Particle)<br>Thermal correction to Energy= 0.283803<br>Thermal correction to Enthalpy= 0.284748<br>Thermal correction to Gibbs Free Energy= 0.225863<br>Sum of electronic and zero-point Energies= -596.683056<br>Sum of electronic and thermal Energies= -596.668533<br>Sum of electronic and thermal Enthalpies= -596.667589<br>Sum of electronic and thermal Free Energies= -596.726473
**Name**	**NVP-C7-OTB-RAF-TL**
Cartesian Coordinates	Frequency and Energy
O 0.38840900 -2.05229800 -0.14891800<br>N 0.66614300 0.23125600 -0.13139400<br>C -1.39141700 0.89973700 0.77633500<br>C -0.13692100 1.43593600 0.07163600<br>C -1.49792700 -0.54848800 0.29286400<br>C -0.06638300 -0.94145700 -0.00847500<br>C 1.99910900 0.24250200 -0.44495400<br>C 2.72326900 1.38434200 -0.60093200<br>H -2.27249800 1.49651700 0.54680200<br>H -1.23323000 0.91866300 1.85598400<br>H -0.36959200 1.89108100 -0.89708700<br>H 0.42375500 2.14675900 0.67843300<br>H -2.05972200 -0.62687400 -0.64366000<br>H -1.93878600 -1.23944100 1.00955000<br>H 2.44713500 -0.74337300 -0.47997900<br>H 3.76102100 1.30141400 -0.89296000<br>H 2.24967300 2.34508800 -0.74011500<br>O 2.88006700 1.97944200 1.41342500<br>C 4.03083000 1.52787200 2.06518800	Zero-point correction= 0.269954 (Hartree/Particle)<br>Thermal correction to Energy= 0.284372<br>Thermal correction to Enthalpy= 0.285316<br>Thermal correction to Gibbs Free Energy= 0.227140<br>Sum of electronic and zero-point Energies= -596.674949<br>Sum of electronic and thermal Energies= -596.660531<br>Sum of electronic and thermal Enthalpies= -596.659587<br>Sum of electronic and thermal Free Energies= -596.717763

C	3.83018800	1.98646300	3.52265600	
C	5.29490400	2.17785500	1.49508400	
C	4.14793100	0.00024800	2.02996700	
H	2.94209900	1.51395600	3.94705900	
H	3.70416500	3.07036800	3.56296500	
H	4.70069400	1.71094500	4.12398500	
H	5.19823800	3.26601600	1.51723500	
H	6.17959200	1.89376200	2.07200500	
H	5.45735500	1.86844300	0.45958500	
H	3.20774700	-0.45635000	2.34912100	
H	4.94809400	-0.34238900	2.69201500	
H	4.37663400	-0.34595400	1.01872000	
**Name**				**NVP-C7-OCM-RAF-IP**
Cartesian Coordinates				Frequency and Energy
O	-1.74812600	-0.35318600	-5.36624300	Zero-point correction= 0.322758 (Hartree/Particle)
N	-0.60879200	1.56332000	-5.92830000	Thermal correction to Energy= 0.340328
C	-2.39643000	2.96106100	-6.53424500	Thermal correction to Enthalpy= 0.341272
C	-0.86831500	2.84326000	-6.59425500	Thermal correction to Gibbs Free Energy= 0.274243
C	-2.87823800	1.50970700	-6.48781300	Sum of electronic and zero-point Energies= -788.342427
C	-1.73263600	0.76377500	-5.84887100	Sum of electronic and thermal Energies= -788.324858
C	0.63170900	1.14151000	-5.52324500	Sum of electronic and thermal Enthalpies= -788.323913
C	1.75862600	1.89007300	-5.63573200	Sum of electronic and thermal Free Energies= -788.390943
H	-2.79091600	3.51604000	-7.38330800	
H	-2.68458600	3.47625700	-5.61642800	
H	-0.49250800	2.79937400	-7.62100300	
H	-0.36075000	3.64908300	-6.06455500	
H	-3.01097000	1.09056600	-7.49104700	
H	-3.79834700	1.35005700	-5.92723700	
H	0.63385500	0.16822700	-5.04626500	
H	2.70470300	1.45003400	-5.34921300	
H	1.78465600	2.80164300	-6.21725600	
O	1.48551800	3.11002500	-3.84814600	
C	2.33552600	2.64683000	-2.84192600	
C	3.80747000	2.78129100	-3.24839300	
C	2.07181800	3.60671200	-1.65749100	
C	1.96457700	1.22120200	-2.41809200	
C	4.79712000	1.93000800	-2.75267100	
C	4.18824700	3.81123500	-4.11334800	
C	6.13232300	2.09937500	-3.11766900	
C	5.51938800	3.98252500	-4.47922000	
C	6.49897900	3.12451800	-3.98299100	
H	1.03192700	3.51862000	-1.33444600	
H	2.72952500	3.34678900	-0.82430600	
H	2.27032600	4.63846900	-1.95513800	
H	0.88256900	1.17143000	-2.27739900	
H	2.44456600	0.94317800	-1.47749200	
H	2.25366000	0.49617700	-3.18290100	
H	4.53733700	1.12251600	-2.07789200	
H	3.42852300	4.47687200	-4.50767600	
H	6.88475600	1.42417000	-2.72474000	
H	5.79304700	4.78604700	-5.15455400	
H	7.53668600	3.25429100	-4.26933300	
**Name**				**NVP-C7-OCM-RAF-TL**
Cartesian Coordinates				Frequency and Energy

O	-1.58086600	-0.46790800	-5.53067200	Zero-point correction= 0.323436 (Hartree/Particle)
N	-0.53220000	1.53289500	-5.96635700	Thermal correction to Energy= 0.340977
C	-2.39475400	2.93406800	-6.23559600	Thermal correction to Enthalpy= 0.341921
C	-0.87343500	2.87424800	-6.43608000	Thermal correction to Gibbs Free Energy= 0.274669
C	-2.84047900	1.47379600	-6.34118200	Sum of electronic and zero-point Energies= -788.335367
C	-1.63018400	0.68641200	-5.88390900	Sum of electronic and thermal Energies= -788.317826
C	0.74008400	1.12598400	-5.66570500	Sum of electronic and thermal Enthalpies= -788.316882
C	1.83242700	1.92774200	-5.77672100	Sum of electronic and thermal Free Energies= -788.384134
H	-2.87504500	3.58516800	-6.96389800	
H	-2.61098100	3.31545300	-5.23620700	
H	-0.58990200	2.97954800	-7.48875800	
H	-0.33533200	3.61437800	-5.84362800	
H	-3.04452500	1.18220600	-7.37664600	
H	-3.71112200	1.21621500	-5.74034900	
H	0.79200800	0.12275500	-5.25931400	
H	2.80771500	1.51755400	-5.55003400	
H	1.80496900	2.85688300	-6.32768900	
O	1.52077100	3.14121400	-4.04908300	
C	2.28992600	2.68569800	-2.98222100	
C	3.78933800	2.77852900	-3.29137300	
C	1.96989900	3.67891100	-1.84030500	
C	1.86395800	1.27713900	-2.54772200	
C	4.73053100	1.94486100	-2.68522800	
C	4.24188300	3.75497100	-4.18215400	
C	6.08897800	2.08041400	-2.96530400	
C	5.59649800	3.89285600	-4.46317000	
C	6.52735800	3.05391000	-3.85540000	
H	0.90947600	3.61788800	-1.58638500	
H	2.56750600	3.43407200	-0.95887700	
H	2.20534900	4.69774200	-2.15278400	
H	0.77335400	1.24280000	-2.50870100	
H	2.25035200	1.02239700	-1.55841900	
H	2.21312300	0.52366200	-3.25768600	
H	4.41223500	1.17675900	-1.98986200	
H	3.51318100	4.39936600	-4.66058200	
H	6.80362200	1.41962000	-2.48752100	
H	5.92754100	4.65517300	-5.15973300	
H	7.58356900	3.15770400	-4.07558300	
**Name**				**IP-C2-C7-NVP-C7-NVP-RAF-IP**
Cartesian Coordinates				Frequency and Energy
O	2.63647400	1.69459800	0.87068500	Zero-point correction= 0.390740 (Hartree/Particle)
N	4.81932900	0.99429600	0.74787600	Thermal correction to Energy= 0.411495
C	4.59515100	-1.27883500	1.28402400	Thermal correction to Enthalpy= 0.412439
C	5.62804300	-0.15203000	1.16168200	Thermal correction to Gibbs Free Energy= 0.340430
C	3.29465300	-0.54535500	1.61851300	Sum of electronic and zero-point Energies= -921.304273
C	3.49428000	0.83493300	1.03702000	Sum of electronic and thermal Energies= -921.283518
C	5.36532000	2.21727400	0.37832900	Sum of electronic and thermal Enthalpies= -921.282574
C	6.69341000	2.50797700	0.48561200	Sum of electronic and thermal Free Energies= -921.354583
H	4.49766900	-1.78963700	0.32432400	
H	6.11165700	0.07195600	2.11806600	
H	6.39648300	-0.35884400	0.41560700	
H	3.16037800	-0.41894400	2.69801300	
H	2.39426500	-1.00817200	1.21490700	
H	4.63029100	2.96069800	0.09470300	

H 7.04336600 3.44139200 0.06023600<br>H 7.43339600 1.72501500 0.60788800<br>O 4.77699700 1.92081500 3.70199500<br>N 5.86686100 3.80024400 2.94629700<br>C 4.19020100 5.42247200 3.19909300<br>C 5.51146500 5.14251300 2.47653400<br>C 3.57279100 4.03327000 3.36932700<br>C 4.76653900 3.10490600 3.37411400<br>C 7.04781400 3.16159600 2.59623500<br>C 8.32048100 3.93791400 2.46010900<br>H 3.55985700 6.11006400 2.63745800<br>H 4.39886800 5.86119700 4.17715600<br>H 5.39078500 5.11958600 1.38674600<br>H 6.28952400 5.86145400 2.72873000<br>H 2.94434100 3.75491100 2.51725800<br>H 2.98537200 3.90602000 4.27901400<br>H 7.10925700 2.15868900 3.00452600<br>H 9.00314900 3.35028500 1.83697400<br>H 8.14636700 4.88558000 1.94160000<br>O 9.37204100 2.98772300 4.44192500<br>C 9.06335500 4.22724600 3.78824100<br>C 8.24783000 5.11046300 4.72700300<br>C 10.40677200 4.87041900 3.47936900<br>H 8.03516500 6.07652000 4.26186900<br>H 7.29800500 4.63626300 4.99023200<br>H 8.81158700 5.28753800 5.64655600<br>H 10.26453900 5.81864000 2.95590200<br>H 11.00945100 4.21121300 2.84937600<br>H 10.95341100 5.06359000 4.40585900<br>H 8.54756100 2.58334000 4.73907200<br>H 4.88258100 -2.00981600 2.03791900	
**Name**	**TL-C5-NVP-C7-NVP-RAF-IP**
Cartesian Coordinates	Frequency and Energy
O 1.76840100 -3.29178500 0.30100000<br>N 1.75970000 -1.01810700 -0.01454800<br>C -0.56951200 -0.61546100 -0.12126700<br>C 0.80602100 0.05646500 -0.28165400<br>C -0.29135600 -2.10943300 -0.30689300<br>C 1.17629000 -2.25959600 0.03802100<br>C 3.05619700 -0.78718100 0.43147300<br>C 3.78149800 0.41100200 -0.09621500<br>H -1.29562600 -0.22261300 -0.83173000<br>H -0.94425200 -0.44169100 0.88877800<br>H 0.96217000 0.45643100 -1.28908700<br>H 0.95316400 0.86706100 0.43547600<br>H -0.43406400 -2.44325600 -1.33934900<br>H -0.89185800 -2.75235300 0.33800200<br>H 3.61732400 -1.71054500 0.53273700<br>H 4.69867900 0.55303800 0.48306900<br>H 3.18251600 1.31838500 0.03632800<br>C 6.87493100 2.77348400 -2.48903300<br>C 6.23588200 1.61092200 -2.06868200<br>C 4.84410700 1.50596900 -2.10459200<br>C 4.10563800 2.59687400 -2.56925800	Zero-point correction= 0.412491 (Hartree/Particle)<br>Thermal correction to Energy= 0.433990<br>Thermal correction to Enthalpy= 0.434934<br>Thermal correction to Gibbs Free Energy= 0.358849<br>Sum of electronic and zero-point Energies= -998.449828<br>Sum of electronic and thermal Energies= -998.428329<br>Sum of electronic and thermal Enthalpies= -998.427385<br>Sum of electronic and thermal Free Energies= -998.503470

C	4.73976000	3.76117200	-2.99043100	
C	6.12816100	3.85300000	-2.95098600	
H	7.95690700	2.83501600	-2.45906300	
H	6.82345500	0.77021600	-1.71241300	
H	3.02219300	2.52911400	-2.60613100	
H	4.15054400	4.59574900	-3.35331800	
H	6.62453100	4.75793400	-3.28151200	
C	4.14879100	0.27119600	-1.59205500	
H	4.79109000	-0.60419000	-1.72028300	
H	3.23614500	0.08831100	-2.16690000	
O	-1.25523700	-1.32522000	3.19534600	
N	0.97217800	-1.88637200	3.15630300	
C	0.83840900	-4.22289500	3.36015400	
C	1.82320200	-3.04620500	3.41036400	
C	-0.50915100	-3.59999000	3.73275600	
C	-0.36728600	-2.14648500	3.32810600	
C	1.45974600	-0.61231400	2.90646400	
C	2.76757000	-0.33991900	2.62627500	
H	1.13795500	-5.03120600	4.02567600	
H	0.80586200	-4.60273900	2.33820400	
H	2.30498500	-2.93697200	4.38856400	
H	2.58645300	-3.12625700	2.63608200	
H	-0.69179400	-3.62123500	4.81223700	
H	-1.36971400	-4.05069300	3.23963000	
H	0.68189000	0.13905400	2.84579900	
H	3.06219800	0.70049300	2.56004600	
H	3.55236100	-1.04447600	2.87451900	
**Name**				**TBO-C7-NVP-C7-NVP-RAF-IP**
Cartesian Coordinates				Frequency and Energy
O	1.81375500	-3.33301800	0.14557100	Zero-point correction= 0.419093 (Hartree/Particle)
N	1.91106700	-1.04454500	0.01463600	Thermal correction to Energy= 0.441188
C	-0.38897600	-0.51048300	-0.09342900	Thermal correction to Enthalpy= 0.442132
C	1.01898600	0.09304600	-0.22122600	Thermal correction to Gibbs Free Energy= 0.366169
C	-0.19052800	-2.00895600	-0.33883400	Sum of electronic and zero-point Energies= -960.561447
C	1.26856900	-2.24646200	-0.02493200	Sum of electronic and thermal Energies= -960.539352
C	3.22192800	-0.90381400	0.44886200	Sum of electronic and thermal Enthalpies= -960.538408
C	3.97365000	0.31683000	0.03314600	Sum of electronic and thermal Free Energies= -960.614372
H	-1.08681800	-0.05248500	-0.79279400	
H	-0.75960900	-0.35061300	0.92029300	
H	1.21235100	0.50864500	-1.21431700	
H	1.20255200	0.86960700	0.52347800	
H	-0.36359100	-2.29433600	-1.38121900	
H	-0.81202800	-2.64706800	0.29260400	
H	3.76324200	-1.84257500	0.47719300	
H	4.92365200	0.34002200	0.57716100	
H	3.41421600	1.22271700	0.28852600	
O	-1.09881500	-1.03837300	3.31909400	
N	1.03320300	-1.87525600	3.13817400	
C	0.61495100	-4.18532600	3.24383700	
C	1.73875700	-3.14540300	3.32900800	
C	-0.63462500	-3.41837400	3.68121200	
C	-0.31326700	-1.97853200	3.35434900	
C	1.67722000	-0.66107100	2.93963500	
C	3.00954700	-0.54796900	2.66551200	

H	0.82343700	-5.05589200	3.86366300	
H	0.50756400	-4.51190300	2.20816900	
H	2.23171800	-3.13868300	4.30685400	
H	2.48869200	-3.27994200	2.55006700	
H	-0.79693100	-3.47553700	4.76295400	
H	-1.55440500	-3.72737000	3.18486600	
H	1.00802400	0.19037200	2.91681300	
H	3.43332800	0.44931900	2.64927000	
H	3.69519300	-1.36247100	2.86726400	
O	4.20743600	0.25461400	-1.37401500	
C	4.66841700	1.46781600	-2.00937000	
C	5.09975500	1.01868800	-3.39905100	
H	5.42309100	1.87673300	-3.99295500	
H	5.92813400	0.30908400	-3.33004400	
H	4.26677300	0.53366400	-3.91447000	
C	3.52145500	2.47180100	-2.11487800	
H	2.68842800	2.03623600	-2.67260000	
H	3.16152500	2.78764200	-1.13264400	
H	3.86012100	3.36558700	-2.64543600	
C	5.85336200	2.06674700	-1.25503600	
H	6.63230100	1.31380600	-1.10568500	
H	6.27704800	2.88824700	-1.83765600	
H	5.55784200	2.46578500	-0.28165100	
**Name**				**TBO-C7-NVP-C7-NVP-RAF-TL**
Cartesian Coordinates				Frequency and Energy
O	1.81476800	-3.32878500	0.28140000	Zero-point correction= 0.419832 (Hartree/Particle)
N	1.91821200	-1.05010000	0.03200900	Thermal correction to Energy= 0.441970
C	-0.37721000	-0.53198000	-0.16138000	Thermal correction to Enthalpy= 0.442914
C	1.03374400	0.07082200	-0.27382900	Thermal correction to Gibbs Free Energy= 0.366611
C	-0.16711500	-2.03562000	-0.35951900	Sum of electronic and zero-point Energies= -960.549453
C	1.27994000	-2.26330100	0.02854900	Sum of electronic and thermal Energies= -960.527315
C	3.22514500	-0.89868500	0.46718600	Sum of electronic and thermal Enthalpies= -960.526371
C	3.96827200	0.32413100	0.03977200	Sum of electronic and thermal Free Energies= -960.602674
H	-1.06151400	-0.09486300	-0.88726500	
H	-0.77112300	-0.34915300	0.83988500	
H	1.25891300	0.45111200	-1.27475000	
H	1.18998300	0.87754400	0.44594100	
H	-0.29100300	-2.34772400	-1.40106000	
H	-0.81858600	-2.65773500	0.25594000	
H	3.76679500	-1.83715100	0.49314200	
H	4.93510200	0.33656300	0.55603500	
H	3.42086000	1.22852600	0.33224600	
O	-1.10124300	-1.05011400	3.20001500	
N	1.04245600	-1.87537600	3.14535800	
C	0.62624900	-4.18301300	3.26853700	
C	1.74451400	-3.13708500	3.37359600	
C	-0.64008100	-3.41628600	3.65689400	
C	-0.32094100	-1.97727500	3.30494600	
C	1.68124600	-0.66053700	2.95429900	
C	3.01487400	-0.53861200	2.68467300	
H	0.81849900	-5.04746800	3.90234200	
H	0.56160700	-4.51593900	2.23162200	
H	2.21411200	-3.11684500	4.36359000	
H	2.50789900	-3.28521700	2.61002000	

H	-0.83237400	-3.45269300	4.73434100	
H	-1.54492000	-3.74235300	3.14523700	
H	1.00113100	0.18215000	2.92744000	
H	3.43672600	0.45943500	2.68208700	
H	3.70320300	-1.34812700	2.89552100	
O	4.14951500	0.28475500	-1.36920900	
C	4.64282500	1.48054400	-1.99767500	
C	4.99301600	1.03591200	-3.41187000	
H	5.33205600	1.88476400	-4.01033400	
H	5.78494100	0.28421800	-3.38626600	
H	4.11665300	0.59612100	-3.89309900	
C	3.54675600	2.54636300	-2.04060000	
H	2.67108400	2.16413100	-2.57040400	
H	3.24084300	2.86250600	-1.04035600	
H	3.90639400	3.43340600	-2.56840300	
C	5.88836500	2.00912200	-1.28674400	
H	6.63022200	1.21383300	-1.17570500	
H	6.33494000	2.81346700	-1.87586900	
H	5.65594800	2.41316600	-0.29831600	
**Name**				**CMO-C7-NVP-C7-NVP-RAF-IP**
Cartesian Coordinates				Frequency and Energy
O	1.88265800	-3.26390200	0.11952200	Zero-point correction= 0.472548 (Hartree/Particle)
N	1.86974100	-0.97340500	-0.01008600	Thermal correction to Energy= 0.497812
C	-0.45495100	-0.54915400	-0.08553900	Thermal correction to Enthalpy= 0.498756
C	0.92134300	0.12209100	-0.22385600	Thermal correction to Gibbs Free Energy= 0.414608
C	-0.18803300	-2.03557600	-0.33896500	Sum of electronic and zero-point Energies= -1152.217043
C	1.28420500	-2.20461900	-0.04259000	Sum of electronic and thermal Energies= -1152.191779
C	3.17902600	-0.77333400	0.40279100	Sum of electronic and thermal Enthalpies= -1152.190834
C	3.86827800	0.48046300	-0.02083000	Sum of electronic and thermal Free Energies= -1152.274983
H	-1.18021900	-0.12241300	-0.77668600	
H	-0.82395400	-0.41182200	0.93202200	
H	1.08248500	0.55664100	-1.21457700	
H	1.07715200	0.89878100	0.52731200	
H	-0.35836200	-2.32481400	-1.38074500	
H	-0.77186200	-2.70468600	0.29611800	
H	3.76311200	-1.68622900	0.42260600	
H	4.82438100	0.54444700	0.50766800	
H	3.27261000	1.36041000	0.24182200	
O	-1.04561300	-1.29379200	3.32142300	
N	1.15456800	-1.92681800	3.13525600	
C	0.95549300	-4.26714600	3.25580600	
C	1.97823900	-3.12517300	3.31313200	
C	-0.35690700	-3.61643300	3.69830800	
C	-0.17480600	-2.15527700	3.35965500	
C	1.67944900	-0.65916800	2.92373700	
C	2.98994000	-0.42424300	2.62426800	
H	1.25182100	-5.10491100	3.88496600	
H	0.86780500	-4.61752700	2.22636800	
H	2.49197500	-3.06798900	4.27845500	
H	2.71917000	-3.19225700	2.51676600	
H	-0.50584900	-3.68053200	4.78152800	
H	-1.24701100	-4.01399900	3.21105500	
H	0.93432000	0.12691500	2.91298000	
H	3.31913000	0.60791200	2.59670600	

H	3.75280500	-1.17081500	2.81095500	
O	4.08227300	0.42272300	-1.43248100	
C	4.51208900	1.63114900	-2.07693600	
C	3.33463800	2.60695200	-2.17369100	
H	2.51424900	2.15077900	-2.73269500	
H	2.97687100	2.88895100	-1.18073500	
H	3.63642900	3.52273700	-2.68580800	
C	5.68629000	2.26420800	-1.32506100	
H	6.48866800	1.53419900	-1.18877700	
H	6.08018800	3.10862700	-1.89291500	
H	5.37334900	2.63790400	-0.34735600	
C	4.96052500	1.22108500	-3.47946200	
C	4.96393600	-0.11062400	-3.89542300	
C	5.38621800	2.20237100	-4.38282400	
C	5.38259800	-0.45302000	-5.18135300	
H	4.63933500	-0.88571800	-3.21411000	
C	5.80405300	1.86143900	-5.66404200	
H	5.39563000	3.24609600	-4.08674200	
C	5.80397800	0.52842600	-6.07039900	
H	5.37746600	-1.49448300	-5.48415400	
H	6.13070400	2.63859500	-6.34626200	
H	6.12907900	0.26087700	-7.06954100	
**Name**				**CMO-C7-NVP-C7-NVP-RAF-TL**
Cartesian Coordinates				Frequency and Energy
O	1.94554800	-3.26880300	0.22865700	Zero-point correction= 0.473376 (Hartree/Particle)
N	1.91410300	-0.98442300	0.01330500	Thermal correction to Energy= 0.498567
C	-0.40931300	-0.59907600	-0.14846600	Thermal correction to Enthalpy= 0.499511
C	0.96229200	0.08794600	-0.26387100	Thermal correction to Gibbs Free Energy= 0.415597
C	-0.11295300	-2.08315300	-0.38005200	Sum of electronic and zero-point Energies= -1152.205786
C	1.34719100	-2.23303700	-0.00378300	Sum of electronic and thermal Energies= -1152.180595
C	3.21406200	-0.76332100	0.43865800	Sum of electronic and thermal Enthalpies= -1152.179651
C	3.88933200	0.49581500	0.00542400	Sum of electronic and thermal Free Energies= -1152.263565
H	-1.12690100	-0.18856200	-0.85747900	
H	-0.80068700	-0.46100900	0.86100600	
H	1.15276400	0.49829600	-1.26021700	
H	1.07926800	0.88909100	0.46979900	
H	-0.22297800	-2.37811400	-1.42818400	
H	-0.72440800	-2.75549000	0.22310500	
H	3.80567700	-1.67131700	0.45987600	
H	4.85438500	0.56353900	0.51919800	
H	3.29370300	1.37142200	0.28930000	
O	-1.04875800	-1.26830700	3.21316600	
N	1.15554100	-1.91064700	3.13041700	
C	0.93474800	-4.24706500	3.23537200	
C	1.96342600	-3.11179700	3.33287100	
C	-0.38698300	-3.59154100	3.64270900	
C	-0.19312900	-2.12794000	3.30187500	
C	1.68774300	-0.64585100	2.94135700	
C	3.00333100	-0.41107700	2.65917000	
H	1.20604000	-5.09485500	3.86273900	
H	0.88521200	-4.57986400	2.19775700	
H	2.44837700	-3.06183800	4.31428300	
H	2.72251600	-3.18830600	2.55437900	
H	-0.56558000	-3.65329600	4.72131700	

H	-1.26614700	-3.98759500	3.13574100	
H	0.93928700	0.13707100	2.92636500	
H	3.33925500	0.61909100	2.65351500	
H	3.76050100	-1.15946800	2.85936700	
O	4.07203900	0.45084600	-1.40456100	
C	4.51378600	1.64817900	-2.05233400	
C	3.35545800	2.64999800	-2.12500500	
H	2.51778400	2.21238800	-2.67153900	
H	3.01909000	2.93822600	-1.12609200	
H	3.66366000	3.56125400	-2.64120800	
C	5.71654800	2.25708400	-1.32445400	
H	6.50366600	1.50892500	-1.20274500	
H	6.12305000	3.08896500	-1.90140100	
H	5.43351100	2.64252900	-0.34193800	
C	4.92433900	1.22938800	-3.46378100	
C	4.88268200	-0.10297100	-3.87254600	
C	5.35654400	2.19558800	-4.37804600	
C	5.26628100	-0.45978200	-5.16410200	
H	4.54887300	-0.85936800	-3.17542900	
C	5.73913000	1.83994000	-5.66564300	
H	5.39880100	3.23981600	-4.08658400	
C	5.69561300	0.50669800	-6.06497100	
H	5.22759500	-1.50127700	-5.46303700	
H	6.07213000	2.60492600	-6.35787000	
H	5.99365900	0.22703200	-7.06880500	
**Name**				**IP-C2-(C7-NVP)$_3$-RAF-IP**
Cartesian Coordinates				Frequency and Energy
O	2.67557200	1.19086200	1.17012100	Zero-point correction= 0.541036 (Hartree/Particle)
N	4.87832000	0.80115400	0.65262000	Thermal correction to Energy= 0.569564
C	5.07980700	-1.45453300	1.28371700	Thermal correction to Enthalpy= 0.570508
C	5.90150500	-0.22317700	0.88149400	Thermal correction to Gibbs Free Energy= 0.479549
C	3.81534300	-0.85677700	1.90408900	Sum of electronic and zero-point Energies= -1285.177158
C	3.67607500	0.47978300	1.21159700	Sum of electronic and thermal Energies= -1285.148631
C	5.10563400	1.98136300	-0.04909300	Sum of electronic and thermal Enthalpies= -1285.147686
C	6.44557300	2.63493700	0.07338800	Sum of electronic and thermal Free Energies= -1285.238645
H	4.81864000	-2.02584800	0.39060300	
H	6.57546900	0.09400500	1.68333200	
H	6.48539100	-0.37755000	-0.02788800	
H	3.93883700	-0.65746300	2.97428800	
H	2.91636000	-1.45775600	1.76979900	
H	4.23454400	2.62721400	-0.04813000	
H	6.48023900	3.48276800	-0.61933400	
H	7.25083000	1.95202000	-0.21690300	
O	4.94066200	2.30185600	3.57850500	
N	5.60927500	3.86950300	2.03928400	
C	4.05759500	5.57887800	2.43661600	
C	5.09923900	5.09239200	1.42045900	
C	3.58500200	4.28807800	3.10815000	
C	4.76918300	3.35469100	2.96325100	
C	6.74569900	3.12600800	1.51005900	
C	8.05207700	3.92053400	1.54209700	
H	3.25448100	6.13952100	1.96082300	
H	4.54265900	6.22299700	3.17319500	
H	4.64514700	4.86563600	0.44857300	

H 5.90304500 5.81356500 1.26591400<br>H 2.74725600 3.82854400 2.57278500<br>H 3.30365700 4.39465100 4.15549000<br>H 6.83975800 2.24906100 2.15417300<br>H 8.77675600 3.37593800 0.92731500<br>H 7.90545300 4.89489500 1.06486900<br>O 9.02772500 2.85297700 3.49846300<br>C 8.70143000 4.12876500 2.92064500<br>C 7.82289000 4.91260400 3.89356800<br>C 10.03587000 4.83785000 2.72967600<br>H 7.46893600 5.84103900 3.43677200<br>H 6.95365100 4.32721000 4.20152800<br>H 8.39986400 5.16247800 4.78800900<br>H 9.88218300 5.82864600 2.29562700<br>H 10.68269200 4.26219200 2.06239400<br>H 10.54080700 4.95407500 3.69168500<br>H 8.21551200 2.44004600 3.81574500<br>H 5.62937900 -2.10358400 1.96359100<br>O 7.38417200 4.05164000 -4.33870200<br>N 7.07152600 2.06489500 -3.23140100<br>C 9.27802200 1.26940900 -3.12314600<br>C 7.81825100 0.84286500 -2.92671000<br>C 9.19271800 2.40688200 -4.14246500<br>C 7.80805600 2.97534300 -3.93322600<br>C 5.71099100 2.22178300 -2.98535500<br>C 4.97921500 1.36777800 -2.21902800<br>H 9.90128100 0.43989400 -3.45277500<br>H 9.67470400 1.64843400 -2.17899600<br>H 7.51457500 0.04984300 -3.61815900<br>H 7.61222700 0.51330900 -1.90762900<br>H 9.24034500 2.03881100 -5.17296100<br>H 9.94883500 3.18223700 -4.02339000<br>H 5.29885400 3.14186900 -3.38085800<br>H 3.90413700 1.49438300 -2.19790800<br>H 5.35570000 0.38446200 -1.95807400	
**Name**	**TL-C7-(C7-NVP)$_3$-RAF-TL**
Cartesian Coordinates	Frequency and Energy
O 1.25422500 -2.56817200 -0.77159500<br>N 1.41863000 -0.33564200 -0.25968700<br>C -0.79095200 0.36027200 -0.62978400<br>C 0.63052700 0.88431700 -0.36544200<br>C -0.56239200 -1.00419100 -1.28408100<br>C 0.79225000 -1.43949900 -0.75093300<br>C 2.71816100 -0.38740700 0.38644600<br>C 3.71022300 0.57764600 -0.26073400<br>H -1.37439700 1.04642100 -1.24238800<br>H -1.30067600 0.21270600 0.32403900<br>H 0.99963700 1.51238000 -1.18590800<br>H 0.69468300 1.46394000 0.55974300<br>H -0.48787700 -0.93993400 -2.37428200<br>H -1.31696900 -1.74886700 -1.03486500<br>H 3.06023300 -1.41420000 0.23324300<br>H 4.65871600 0.51864100 0.28347900<br>H 3.34931600 1.60687200 -0.14453400	Zero-point correction= 0.562096 (Hartree/Particle)<br>Thermal correction to Energy= 0.591765<br>Thermal correction to Enthalpy= 0.592709<br>Thermal correction to Gibbs Free Energy= 0.495830<br>Sum of electronic and zero-point Energies= -1362.321039<br>Sum of electronic and thermal Energies= -1362.291371<br>Sum of electronic and thermal Enthalpies= -1362.290427<br>Sum of electronic and thermal Free Energies= -1362.387306

C	7.21111000	1.89255800	-2.88001200	
C	6.30000400	0.97414700	-2.36717000	
C	4.93230900	1.25434600	-2.35159500	
C	4.49826400	2.47963100	-2.86228300	
C	5.40491000	3.40164300	-3.37548000	
C	6.76595900	3.11033900	-3.38541500	
H	8.26897800	1.65535300	-2.88894300	
H	6.65213700	0.02358500	-1.97806400	
H	3.43663800	2.70927200	-2.86293700	
H	5.04890300	4.34573700	-3.77218100	
H	7.47390900	3.82511100	-3.78851300	
C	3.95058200	0.28300100	-1.74589000	
H	4.32169900	-0.74048600	-1.85491200	
H	2.99868900	0.33543100	-2.28294000	
O	-1.21577200	-1.79032400	2.11064200	
N	1.05877700	-2.04402000	2.33112600	
C	1.26971400	-4.36532300	2.03517400	
C	2.03062800	-3.11887300	2.50884700	
C	-0.19976100	-4.01854000	2.29020200	
C	-0.23342300	-2.50774800	2.22940400	
C	1.34593200	-0.70600600	2.49252700	
C	2.59728500	-0.12900700	1.91408400	
H	1.59792400	-5.26241700	2.55831300	
H	1.43500800	-4.49227600	0.96524000	
H	2.31303100	-3.17934900	3.56742700	
H	2.92729600	-2.92933400	1.91704700	
H	-0.53592800	-4.31021600	3.29059100	
H	-0.89181300	-4.44049000	1.56195700	
H	0.45007900	-0.10135000	2.49217600	
H	2.60057000	0.94706600	2.11323400	
H	3.49654100	-0.53781000	2.38942400	
O	-1.59314900	-3.07949500	5.39690000	
N	-0.97712000	-0.88330000	5.13216300	
C	-3.07063800	0.15656200	5.00480500	
C	-1.57893700	0.37479500	4.71392600	
C	-3.24734900	-1.35548400	4.84922700	
C	-1.88178800	-1.92120600	5.17313200	
C	0.38853500	-1.11024100	5.18410600	
C	1.33347700	-0.26104600	4.71865900	
H	-3.70302600	0.73402700	4.33216200	
H	-3.29114600	0.45789700	6.03075100	
H	-1.39947500	0.53392800	3.64526300	
H	-1.14911500	1.20392800	5.27988700	
H	-3.45374900	-1.63423000	3.81164200	
H	-4.01282900	-1.79608000	5.48679100	
H	0.64114400	-2.10025100	5.54487400	
H	2.37190800	-0.53126800	4.86430200	
H	1.12152500	0.75846900	4.41549900	
**Name**				**CMO-(C7-NVP)$_3$-RAF-IP**
Cartesian Coordinates				Frequency and Energy
O	1.11190400	-1.63390400	-0.49661000	Zero-point correction= 0.621923 (Hartree/Particle)
N	1.88549900	0.42398800	0.16871000	Thermal correction to Energy= 0.654287
C	-0.05104600	1.72497100	-0.06086000	Thermal correction to Enthalpy= 0.655231
C	1.46598200	1.82361400	0.15174000	Thermal correction to Gibbs Free Energy= 0.554403

C	-0.22418400	0.39774000	-0.80339200	Sum of electronic and zero-point Energies= -1516.098115
C	0.97753300	-0.41765600	-0.36668600	Sum of electronic and thermal Energies= -1516.065750
C	3.16113600	-0.00606800	0.71239900	Sum of electronic and thermal Enthalpies= -1516.064806
C	4.31990300	0.68754500	0.01337800	Sum of electronic and thermal Free Energies= -1516.165634
H	-0.44470000	2.58193500	-0.60553100	
H	-0.54889600	1.66925300	0.90975300	
H	1.96209600	2.35527000	-0.66762200	
H	1.73089100	2.31291600	1.09210800	
H	-0.16184400	0.51988900	-1.88969400	
H	-1.14690800	-0.13311800	-0.57037000	
H	3.22333800	-1.07746800	0.51080500	
H	5.25503800	0.37130500	0.48947100	
H	4.22794100	1.77359700	0.13203500	
O	-0.80118300	-0.78564200	2.93620700	
N	1.41953000	-1.36426700	2.80790000	
C	1.26644400	-3.70046900	2.61941400	
C	2.24484100	-2.56668200	2.94739500	
C	-0.09572600	-3.13527500	3.02906800	
C	0.08473600	-1.63764700	2.92244900	
C	1.92604100	-0.07836700	2.90769000	
C	3.23057000	0.23029200	2.24170500	
H	1.52560700	-4.62044700	3.14113300	
H	1.28198600	-3.89187300	1.54510000	
H	2.61866700	-2.62548900	3.97650500	
H	3.09585100	-2.53528300	2.26688700	
H	-0.34136100	-3.36267900	4.07153200	
H	-0.92685000	-3.46352300	2.40447100	
H	1.14627500	0.67196500	2.84259900	
H	3.48153200	1.27533900	2.44819300	
H	4.04507000	-0.38181400	2.64092000	
O	4.29035400	0.30785900	-1.34825300	
C	5.08685000	1.07012200	-2.26374700	
C	4.44401700	2.44303900	-2.48527900	
H	3.42298700	2.32177000	-2.85536800	
H	4.42269700	3.02141100	-1.55875900	
H	5.01385200	3.01597000	-3.21941000	
C	6.52010200	1.21308000	-1.74515300	
H	6.94320800	0.22986200	-1.52283200	
H	7.14488600	1.69696200	-2.49751100	
H	6.54989700	1.82722400	-0.84202200	
C	5.08227200	0.28446800	-3.57417700	
C	4.41257100	-0.93267200	-3.70438100	
C	5.76305700	0.79400300	-4.68604800	
C	4.42650300	-1.62482300	-4.91547800	
H	3.87673200	-1.34352000	-2.85876400	
C	5.77748100	0.10408100	-5.89268800	
H	6.28970100	1.73990900	-4.61342800	
C	5.10789700	-1.11226700	-6.01289100	
H	3.89969500	-2.56949800	-4.99577500	
H	6.31254600	0.51725300	-6.74068000	
H	5.11834500	-1.65194800	-6.95318000	
O	-0.99448000	-2.02144500	6.28474300	
N	0.03113500	-0.00080000	5.91758000	
C	-1.77536100	1.47476800	6.16551800	
C	-0.34416400	1.38207600	5.62292700	

C	-2.29954800	0.04139900	6.05487900	
C	-1.05179900	-0.80893000	6.11671600	
C	1.31722800	-0.49783900	5.73831500	
C	2.31139000	0.20751800	5.13520500	
H	-2.37263700	2.19507600	5.60897100	
H	-1.74828500	1.78090300	7.21288500	
H	-0.30094500	1.54912200	4.54139400	
H	0.34363400	2.07093000	6.11535300	
H	-2.76868000	-0.15081800	5.08429000	
H	-3.00222300	-0.24776700	6.83615000	
H	1.42956500	-1.53907200	6.01508600	
H	3.30655600	-0.22080300	5.11668300	
H	2.23858500	1.27956600	4.99191700	
**Name**				**CMO-(C7-NVP)$_3$-RAF-TL**
Cartesian Coordinates				Frequency and Energy
O	1.11408800	-1.63936900	-0.49409000	Zero-point correction= 0.622221 (Hartree/Particle)
N	1.87877600	0.42415000	0.16397600	Thermal correction to Energy= 0.654715
C	-0.06313300	1.71604000	-0.07054600	Thermal correction to Enthalpy= 0.655659
C	1.45344900	1.82192200	0.14172500	Thermal correction to Gibbs Free Energy= 0.553935
C	-0.23058500	0.38546700	-0.80836400	Sum of electronic and zero-point Energies= -1516.078384
C	0.97445600	-0.42326100	-0.36850800	Sum of electronic and thermal Energies= -1516.045890
C	3.15619800	0.00134800	0.70903100	Sum of electronic and thermal Enthalpies= -1516.044945
C	4.31218200	0.69963000	0.01009600	Sum of electronic and thermal Free Energies= -1516.146669
H	-0.46051000	2.56938500	-0.61817800	
H	-0.56068900	1.66153500	0.90030000	
H	1.94749400	2.35254300	-0.67964600	
H	1.71622800	2.31591500	1.08024300	
H	-0.16847800	0.50400500	-1.89507800	
H	-1.15113700	-0.14839000	-0.57363000	
H	3.22378900	-1.07004100	0.50916000	
H	5.24794800	0.38737800	0.48740800	
H	4.21506200	1.78539300	0.12808800	
O	-0.80215800	-0.79351500	2.94266800	
N	1.42116900	-1.36106000	2.80964500	
C	1.27954400	-3.69851500	2.62830700	
C	2.25302100	-2.55883400	2.95039700	
C	-0.08426200	-3.13920100	3.04051200	
C	0.08812400	-1.64098400	2.92886000	
C	1.92129800	-0.07238300	2.90499800	
C	3.22412000	0.24081100	2.23792900	
H	1.54497100	-4.61578300	3.15164100	
H	1.29287800	-3.89261900	1.55443900	
H	2.62987000	-2.61277400	3.97867900	
H	3.10202600	-2.52506100	2.26751100	
H	-0.32541700	-3.36472000	4.08443300	
H	-0.91562200	-3.47368100	2.41954400	
H	1.13791600	0.67404500	2.83839300	
H	3.47020600	1.28743900	2.44229900	
H	4.04150400	-0.36663500	2.63836500	
O	4.28473800	0.31926100	-1.35125900	
C	5.09682700	1.06914400	-2.26334600	
C	4.47959500	2.45377600	-2.48440600	
H	3.45680200	2.35197400	-2.85544900	
H	4.46889600	3.03179600	-1.55751800	

H	5.06063600	3.01656000	-3.21759400	
C	6.53103900	1.18616000	-1.74082300	
H	6.93557500	0.19547000	-1.51706600	
H	7.16661300	1.65838700	-2.49154600	
H	6.56954400	1.79998100	-0.83781000	
C	5.08191000	0.28520300	-3.57476400	
C	4.39186400	-0.92021900	-3.70800300	
C	5.77443200	0.78388300	-4.68427900	
C	4.39718200	-1.61137000	-4.91973100	
H	3.84705800	-1.32281600	-2.86417400	
C	5.78047200	0.09485800	-5.89149800	
H	6.31696300	1.72057200	-4.60951000	
C	5.09042100	-1.10969300	-6.01475400	
H	3.85455100	-2.54685000	-5.00226400	
H	6.32510500	0.49944100	-6.73753900	
H	5.09427000	-1.64865700	-6.95550400	
O	-0.98165000	-2.02260500	6.29539300	
N	0.03141600	0.00270400	5.91931600	
C	-1.78300800	1.46864000	6.16652700	
C	-0.35245100	1.38242900	5.62113100	
C	-2.29914900	0.03189600	6.06184100	
C	-1.04636600	-0.81098800	6.12357100	
C	1.31984700	-0.48756100	5.73825100	
C	2.30855100	0.22175000	5.13075800	
H	-2.38565500	2.18358800	5.60886400	
H	-1.75545500	1.77846400	7.21279500	
H	-0.31247600	1.54626600	4.53899200	
H	0.33242100	2.07677800	6.10989900	
H	-2.76939600	-0.16625000	5.09298900	
H	-2.99833600	-0.25871400	6.84570200	
H	1.43877900	-1.52733800	6.01772700	
H	3.30608300	-0.20096900	5.11129500	
H	2.22935900	1.29298600	4.98476700	
**Name**				**TBO-(C7-NVP)$_3$-RAF-IP**
Cartesian Coordinates				Frequency and Energy
O	0.99956500	-2.23709200	0.19305900	Zero-point correction= 0.568119 (Hartree/Particle)
N	1.50184000	-0.03452100	0.61121500	Thermal correction to Energy= 0.598324
C	-0.53890100	0.99422600	0.08581500	Thermal correction to Enthalpy= 0.599268
C	0.93790900	1.29351300	0.37876200	Thermal correction to Gibbs Free Energy= 0.503449
C	-0.51223200	-0.42868900	-0.47742900	Sum of electronic and zero-point Energies= -1324.442491
C	0.73050000	-1.03737200	0.14323800	Sum of electronic and thermal Energies= -1324.412287
C	2.76690100	-0.23900200	1.29441400	Sum of electronic and thermal Enthalpies= -1324.411342
C	3.89583000	0.50273200	0.59597600	Sum of electronic and thermal Free Energies= -1324.507161
H	-0.97697100	1.72265900	-0.59501300	
H	-1.10276200	1.00722000	1.02126500	
H	1.43681400	1.77114200	-0.47154600	
H	1.07454200	1.92557800	1.25955000	
H	-0.37750300	-0.44129800	-1.56395200	
H	-1.38944000	-1.02755200	-0.23376800	
H	2.96413100	-1.31160000	1.23806900	
H	4.81250700	0.37529400	1.18410300	
H	3.66378900	1.57420100	0.55865600	
O	-1.26894900	-1.19133600	3.29127100	
N	1.00243900	-1.51873900	3.41882600	

C	1.11603600	-3.86355500	3.51451000	
C	1.93410000	-2.59276000	3.77207100	
C	-0.33138300	-3.41647600	3.73359500	
C	-0.30037900	-1.93015700	3.45698900	
C	1.36322800	-0.18122900	3.38913600	
C	2.68064400	0.18774600	2.78192800	
H	1.41888700	-4.67809300	4.17062500	
H	1.25532300	-4.17933300	2.47925200	
H	2.21695200	-2.48713600	4.82613600	
H	2.83544600	-2.54206700	3.16093300	
H	-0.65369600	-3.55064700	4.77110500	
H	-1.05788500	-3.90769500	3.08580400	
H	0.51961900	0.46470200	3.17428400	
H	2.80652300	1.27141900	2.86803900	
H	3.51666600	-0.27615700	3.31417700	
O	4.04508900	-0.04007200	-0.70000000	
C	4.83791700	0.71442100	-1.64050100	
C	5.02594200	-0.23662200	-2.81460500	
H	5.58283200	0.25369000	-3.61621400	
H	5.57708400	-1.12614600	-2.49918500	
H	4.05450400	-0.54918400	-3.20629600	
C	4.07383700	1.95891900	-2.08967000	
H	3.10500200	1.67461500	-2.50896600	
H	3.91149000	2.65865400	-1.26641000	
H	4.64270600	2.48334000	-2.86177800	
C	6.18982800	1.08917400	-1.03790000	
H	6.69207400	0.20150300	-0.64328900	
H	6.82460300	1.52837200	-1.81115300	
H	6.08687200	1.82247100	-0.23421800	
O	-1.64167100	-2.01193800	6.73818200	
N	-0.78664500	0.05196300	6.20866100	
C	-2.74289100	1.34469200	6.13657300	
C	-1.26588200	1.34638600	5.72380900	
C	-3.11316500	-0.13979200	6.15523200	
C	-1.79999300	-0.83952000	6.41898000	
C	0.55234700	-0.32275800	6.19316100	
C	1.52464000	0.40784700	5.58467600	
H	-3.35422900	1.93185500	5.45333400	
H	-2.84096300	1.76475800	7.13926300	
H	-1.14055800	1.39967200	4.63707200	
H	-0.69594600	2.15419000	6.18508000	
H	-3.46852000	-0.48661200	5.17917900	
H	-3.85438800	-0.41489000	6.90530800	
H	0.73849200	-1.30743000	6.60432800	
H	2.55344700	0.08626000	5.69572700	
H	1.36432400	1.44215600	5.30242700	
**Name**				**TBO-(C7-NVP)$_3$-RAF-TL**
Cartesian Coordinates				Frequency and Energy
O	1.12148000	-2.37595900	0.42105300	Zero-point correction= 0.569558 (Hartree/Particle)
N	1.55416700	-0.12959500	0.61386400	Thermal correction to Energy= 0.599538
C	-0.52824600	0.76582300	0.03517000	Thermal correction to Enthalpy= 0.600482
C	0.94112900	1.14619200	0.27570300	Thermal correction to Gibbs Free Energy= 0.505675
C	-0.44992000	-0.69292000	-0.42179900	Sum of electronic and zero-point Energies= -1324.423417
C	0.81408400	-1.20914200	0.24669400	Sum of electronic and thermal Energies= -1324.393437

C	2.82691100	-0.24656600	1.29286500	Sum of electronic and thermal Enthalpies= -1324.392493
C	3.91262300	0.54905700	0.58246800	Sum of electronic and thermal Free Energies= -1324.487299
H	-1.01295700	1.42556100	-0.68342100	
H	-1.07030100	0.81468600	0.98230400	
H	1.41033500	1.57165700	-0.61961700	
H	1.05895200	1.85998100	1.09643800	
H	-0.31390500	-0.78280900	-1.50418100	
H	-1.30573900	-1.30099100	-0.13200900	
H	3.08252800	-1.30638600	1.23869000	
H	4.83590800	0.47661400	1.17243600	
H	3.62412300	1.60901600	0.54652500	
O	-1.32201500	-0.78948000	3.34165300	
N	0.90050500	-1.36455200	3.39393000	
C	0.76215900	-3.71266500	3.39939800	
C	1.72247000	-2.54217900	3.65751800	
C	-0.62703900	-3.12188500	3.65742100	
C	-0.44483000	-1.63496100	3.44378400	
C	1.38860400	-0.06865100	3.38780000	
C	2.73544300	0.19084500	2.78283000	
H	0.98861600	-4.56657700	4.03628700	
H	0.85003100	-4.01088200	2.35507900	
H	2.07703300	-2.51111400	4.69521400	
H	2.58393400	-2.56687100	2.98911800	
H	-0.95891100	-3.26201500	4.69096900	
H	-1.40355600	-3.50266500	2.99385400	
H	0.60258600	0.64361500	3.16162300	
H	2.93993000	1.26355100	2.87040100	
H	3.52877900	-0.32440100	3.33458900	
O	4.08485700	0.01458500	-0.70577500	
C	4.88723500	0.76600100	-1.63084800	
C	5.09630700	-0.18967500	-2.79807600	
H	5.65873100	0.29664900	-3.59835500	
H	5.64574700	-1.07402800	-2.46824500	
H	4.13104800	-0.51308600	-3.19367900	
C	4.12764200	2.00768600	-2.09945800	
H	3.17243500	1.71650500	-2.54289800	
H	3.93790500	2.70612900	-1.28071600	
H	4.70958500	2.54012700	-2.85596500	
C	6.23159900	1.14998300	-1.01346700	
H	6.72590600	0.26763000	-0.59840100	
H	6.88110300	1.57647000	-1.78130400	
H	6.12098500	1.89581300	-0.22227000	
O	-1.38278300	-2.07637500	6.84501100	
N	-0.68053400	0.03440300	6.27409200	
C	-2.72499300	1.18145200	6.21022000	
C	-1.25415600	1.28159900	5.78126900	
C	-2.99080600	-0.32484300	6.24960900	
C	-1.63030600	-0.93238000	6.51676200	
C	0.67268100	-0.26066300	6.22304700	
C	1.58901200	0.51063100	5.57187200	
H	-3.38201200	1.71547000	5.52545700	
H	-2.84518600	1.60846600	7.20787900	
H	-1.15140200	1.32074000	4.69232500	
H	-0.73974200	2.13449700	6.22843700	
H	-3.31158600	-0.70472900	5.27485500	

H	-3.71164600	-0.64253900	7.00199700	
H	0.91696400	-1.23175900	6.63541300	
H	2.63612200	0.25011700	5.66924900	
H	1.37264800	1.53969400	5.30921700	
